# *Lactobacillus* species as biomarkers and agents that can promote various aspects of vaginal health

**DOI:** 10.3389/fphys.2015.00081

**Published:** 2015-03-25

**Authors:** Mariya I. Petrova, Elke Lievens, Shweta Malik, Nicole Imholz, Sarah Lebeer

**Affiliations:** ^1^Department of Bioscience Engineering, University of AntwerpAntwerp, Belgium; ^2^Centre of Microbial and Plant Genetics, KU LeuvenLeuven, Belgium

**Keywords:** vaginal microbiota, lactobacilli, bacterial vaginosis, STIs, probiotics

## Abstract

The human body is colonized by a vast number of microorganisms collectively referred to as the human microbiota. One of the main microbiota body sites is the female genital tract, commonly dominated by *Lactobacillus* spp., in approximately 70% of women. Each individual species can constitute approximately 99% of the ribotypes observed in any individual woman. The most frequently isolated species are *Lactobacillus crispatus*, *Lactobacillus gasseri*, *Lactobacillus jensenii* and *Lactobacillus iners*. Residing at the port of entry of bacterial and viral pathogens, the vaginal *Lactobacillus* species can create a barrier against pathogen invasion since mainly products of their metabolism secreted in the cervicovaginal fluid can play an important role in the inhibition of bacterial and viral infections. Therefore, a *Lactobacillus*-dominated microbiota appears to be a good biomarker for a healthy vaginal ecosystem. This balance can be rapidly altered during processes such as menstruation, sexual activity, pregnancy and various infections. An abnormal vaginal microbiota is characterized by an increased diversity of microbial species, leading to a condition known as bacterial vaginosis. Information on the vaginal microbiota can be gathered from the analysis of cervicovaginal fluid, by using the Nugent scoring or the Amsel's criteria, or at the molecular level by investigating the number and type of *Lactobacillus* species. However, when translating this to the clinical setting, it should be noted that the absence of a *Lactobacillus*-dominated microbiota does not appear to directly imply a diseased condition or dysbiosis. Nevertheless, the widely documented beneficial role of vaginal *Lactobacillus* species demonstrates the potential of data on the composition and activity of lactobacilli as biomarkers for vaginal health. The substantiation and further validation of such biomarkers will allow the design of better targeted probiotic strategies.

## Introduction

Understanding the link between the microbiota and our health is the focus of a growing number of research projects and papers, with new insights becoming available every day. The Human Microbiome Project and the European MetaHIT consortium initiated almost one decade ago, aim at detailed characterization of the structure and the composition of the microbiota from various body sites. Primary attention has been focused on the composition and function of the gastrointestinal (GI) microbiota and its relation with health and disease. Nevertheless, in recent years, the microbiota from other body sites, such as the skin, oronasopharyngeal cavity and genital tract, have also gained more attention. An important body site providing a habitat for the development of structured microbial communities is the vaginal tract, which is broadly colonized by microorganisms known as the vaginal microbiota (VMB). Residing at the port of entry of various pathogens causing urogenital and sexually transmitted infections (STIs) in women, there has been an increasing interest in the composition and function of the VMB. Therefore, the VMB has been recognized as an important factor involved in the protection of the host from various bacterial, fungal and viral pathogens. In addition, the VMB of the mother plays an essential role in the initial colonization of new-born babies and therefore the development of a healthy GI and skin microbiota (Dominguez-Bello et al., 2010). It is recognized for a long time that the healthy VMB (which generally refers to lack of symptoms, absence of various infections, and good pregnancy outcome) is dominated mainly by *Lactobacillus* species, of which the presence can be therefore used as a valuable biomarker for evaluating health and disease. However, it is only now since the more wide-spread application of high throughput sequencing approaches possible to provide a balanced insight and perspective on their multifaceted appearance in the VMB and their multifaceted role in vaginal health.

## *Lactobacillus* species as biomarkers for vaginal health in different community groups of VMB

As mentioned above, lactobacilli are dominant species in approximately 70% of women. Because of the limitations of culture-based approaches, the detection and clustering of the VMB in different groups is based in the last few years on culture-independent methods, since culture-dependent methods have some limitations. These molecular techniques include Sanger sequencing of 16S rRNA of bacterial colonies (e.g., Verhelst et al., 2005), terminal restriction fragment length polymorphism (T-RFLP) of 16S rRNA (e.g., Zhou et al., 2010), qPCR (e.g., Jespers et al., 2012; Datcu et al., 2013) and next generation sequencing (NGS) (Forney et al., 2010; Hummelen et al., 2010; Ravel et al., 2011; Martin et al., 2012; Smith et al., 2012; Srinivasan et al., 2012; Drell et al., 2013; Lee et al., 2013). Although different techniques have been used, similar patterns have been observed in most studies. For example, Verhelst et al. (2005) were one of the first to categorize the VMB in a number of different grades or community types based on Gram stain, the isolated dominant species, as well as DNA sequencing of 16S rRNA genes. More specifically, the authors reported the presence of four vaginal grades. Grade I was characterized by a normal microbiota and has been subsequently separated in grade Ia and Iab, in which *L. crispatus* is the most dominant *Lactobacillus* species followed by *L. jensenii*, and Ib, in which *L. iners* and *L. gasseri* are predominant. In addition, the presence of a grade I-like VMB consists mainly of *Bifidobacterium* spp. and some lactobacilli, mainly *L. gasseri*. Grade II represents an intermediate status between grade I and grade III, with the presence of *L. iners, L. gasseri*, *L. crispatus, Atopobium vaginae*, *Gardnerella vaginalis*, *Actinomyces neuii* and *Peptoniphilus*. Grade III is characterized by the presence of BV-associated species (*Prevotella bivia*, *A. vaginae*, *G. vaginalis*, *Bacteroides ureolyticus* and *Mobiluncus curtisii)* and low amounts of *Lactobacillus* species, mainly *L. iners*. Finally, Grade IV is characterized by the presence of a variety of *Streptococcus* spp. (Verhelst et al., 2005). The results observed by Verhelst et al. were further confirmed in Belgian women in follow up studies performed by the same group (El Aila et al., 2009; Santiago et al., 2011).

Hummelen et al. (2010) reported, by using an Illumina-based amplicon sequencing of the V6 region of the 16S rRNA gene, on the presence of eight major clusters in Tanzanian women, with only two of them associated with a normal microbiota and dominated by *L. crispatus* and *L. iners*. The authors also described the presence of four BV-associated clusters, dominated by *P. bivia*, *Lachnospiraceae*, or a mixture of different species. The remaining clusters were characterized as normal, intermediate or BV-associated and were dominated mainly by *G. vaginalis* and *L. iners* (Hummelen et al., 2010).

**Figure 1:**
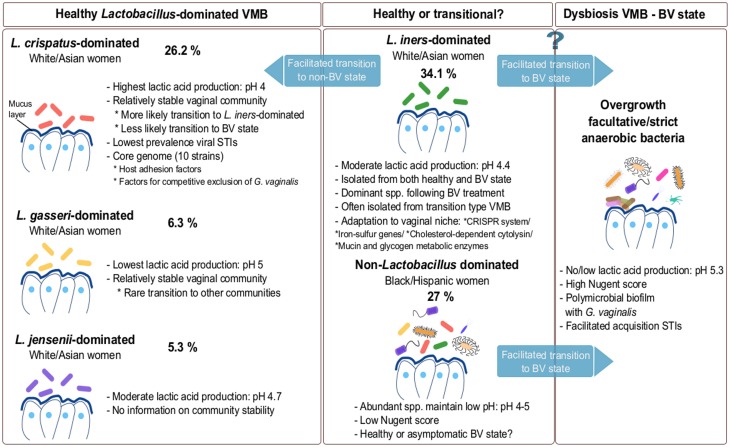
**Composition of VMB during healthy and dysbiotic states**. The vaginal microbiota in healthy adult premenopausal women can be divided into different community groups. The exact number and type of community groups is still under debate (Verhelst et al., 2005; Ravel et al., 2011; Gajer et al., 2012; Santiago et al., 2012). The most commonly isolated dominating species belong to *L. crispatus*, *L. gasseri*, *L. iners* and *L. jensenii*. The vaginal community group dominated by *L. iners* is also often isolated during menstruation and in the transitional microbiota between healthy and BV state or vice-versa. Because this species is often isolated during BV, *L. iners* may not be able to effectively protect against pathogens. Additionally, a non-*Lactobacillus* dominated VMB is also documented in various healthy individuals (Zhou et al., 2004; Ravel et al., 2011; Santiago et al., 2012). For each compositional state, we have added an example of their abundance based on the study of Ravel et al. (2011). However, these numbers are only exemplary and should be considered with caution, as they clearly depend on the study population (size and characteristics) and they certainly need to be substantiated in further studies.

Using pyrosequencing of the V1–V2 hypervariable regions of 16S rRNA genes, Ravel et al. (2011) suggested that the VMB can be divided in five major microbial communities based on samples from four ethnic groups—white, black, Hispanic and Asian women in North America. According to the authors, microbial communities belonging to group I (26.2%), II (6.3%), III (34.1%), and V (5.3%) are dominated by *L. crispatus*, *L. gasseri*, *L. iners*, and *L. jensenii*, respectively, and were isolated mainly from white and Asian women (Figure 1), while group IV was characterized by diverse species (see Non-*Lactobacillus* dominated healthy VMB). Of interest, Smith et al. (2012) observed that the cervical microbiota clusters in six distinct community types of which clusters I–IV are similar to the vaginal community types reported by Ravel and co-workers. The authors were able to detect two additional community types labeled as VI and VII, but were not able to detect a *L. jensenii* dominated microbiota, community type V as described by Ravel and co-workers. Type VI was characterized by the presence of *G. vaginalis*, whereas type VII showed high, approximately even proportions of *G. vaginalis* and *Lactobacillus* spp. An additional cluster designated IIIb, was characterized by a predominance of *L. iners* (Smith et al., 2012). Srinivasan et al. (2012) observed that women without BV have vaginal bacterial communities dominated either by *L. crispatus* or *L. iners* based on 16S rRNA gene PCR and pyrosequencing. Other abundant lactobacilli in women without BV in their study included *L. jensenii* and *L. gasseri*, while five women without BV had different dominant bacteria including *A. vaginae*, *Leptotrichia amnionii*, *Prevotella amii*, a first phylogenetically-distinct group of DNA sequences representing BV-associated bacteria (BVAB1) and *Fusobacterium gonidiaformans* (Srinivasan et al., 2012). Therefore, the actual number of vaginal community types is still under discussion and is driven by the technology used, the sequencing depth, the number of samples of each type, the computational choice made, as well as the underlying data structures. However taken together, the VMB appears to be mainly dominated by *L. crispatus* and *L. iners*, while clusters dominated by *L. jensenii* and *L. gasseri* appear less common (Figure 1). Of note, *L. iners* is present in almost all women, including those with dysbiosis, while *L. crispatus* is typically isolated from healthy women (see *L. crispatus*—dominated microbiota and *L. iners*—harmful or beneficial for vaginal health?). Furthermore, most studies also report clusters without clear dominant species, but instead a proportion of multiple *Lactobacillus* species. Other *Lactobacillus* species, such as *Lactobacillus rhamnosus* (Pascual et al., 2008b), *Lactobacillus plantarum* (Martin et al., 2008), *Lactobacillus vaginalis* (Srinivasan et al., 2012), *Lactobacillus salivarius* (Gustafsson et al., 2011) and *Lactobacillus coleohominis* (Srinivasan et al., 2012) can also be detected occasionally. A key challenge for future studies on the VMB composition is to integrate and further substantiate the findings of the studies using differing sequencing approaches. Hopefully, this will result in the identification of clinically relevant microbiota clusters that could be good biomarkers of the VMB state. This might be, as compared to the fecal enterotypes (Siezen and Kleerebezem, 2011), more difficult than initially anticipated given the fact that human microbial communities at different body sites appear in a continuum of different compositions, as we will also highlight below for the VMB.

### *L. crispatus*—dominated microbiota

According to the study of Ravel et al. (2011) mentioned above, *L*. *crispatus* represents the dominant species of the vaginal bacterial community group I and is isolated mainly from white and Asian women. *L*. *crispatus* and *L*. *iners* are also detected in group IV that is not dominated by *Lactobacillus* species. The identification of *L*. *crispatus* as the most frequently occurring species, has also been reported in other populations such as Turkish, Swedish, Mexican, Belgian, Japanese, American and Canadian women (Kilic et al., 2001; Vasquez et al., 2002; Verhelst et al., 2005; Ravel et al., 2011; Martinez-Pena et al., 2013; Chaban et al., 2014). Frequent occurrence/dominance of this species has also been reported in pregnant women based on a combined approach of microbiological methods and genus-specific, multiplex and species-specific PCR (Kiss et al., 2007). Apart from its occurrence in the vagina, the species has been reported to be present in the rectum of healthy individuals and this colonization appears to be associated with a decreased risk of BV (El Aila et al., 2009).

Recently, a comparative genome analysis of the most abundant vaginal *Lactobacillus* species explored adaptation mechanisms of vaginal lactobacilli in the vaginal ecosystem (Mendes-Soares et al., 2014). *L*. *crispatus* has on average the largest genome with the highest number of proteins unlike the other vaginal strains studied. The *L*. *crispatus* strains were found to have a unique DNA polymerase, bacteriocin and toxin-antitoxin systems and genes encoding mobile genetic elements, especially transposases that contribute to its large genome size. Interestingly, lysogeny of phage particles was observed recently in most of the vaginal isolates of *L*. *crispatus*, which could explain the large number of genes encoding mobile genetic elements (Damelin et al., 2011).

van de Wijgert et al. (2014) highlight in their systematic review that longitudinal studies have indicated that a *L*. *crispatus*-dominated VMB is more likely to transition to a *L*. *iners*-dominated VMB and less likely to a dysbiotic state such as BV and vice-versa. Another study showed that women with a VMB dominated by *L*. *crispatus* have the lowest prevalence of human immunodeficiency virus (HIV), herpes simplex virus type 2 (HSV-2) and human papillomavirus (HPV) (Borgdorff et al., 2014). In addition, it has been reported that detection of *L. crispatus* was associated with a 35% lower risk of HIV-1 RNA shedding (Mitchell et al., 2013).

The presence of BV and loss of lactobacilli appears one of the risk factors of preterm delivery (Donders et al., 2009). For instance, 56% of women who delivered at term (*n* = 98) were reported in another study to be colonized by two or more species of lactobacilli which included *L*. *crispatus* (Petricevic et al., 2014). The exact mechanisms by which lactobacilli could prevent preterm delivery are not known, but pathogen inhibition could be involved. For instance, *Escherichia coli* infection is a frequent cause of preterm birth (Carey and Klebanoff, 2005) and *L*. *crispatus* ATCC 33197 has been shown to contribute to the inhibitory activity of vaginal secretions against *E*. *coli* (Ghartey et al., 2014). The exact inhibitory compounds remain to be identified, but many clinical isolates of *L*. *crispatus* produce H_2_O_2_ *in vitro*, which has been proposed by Antonio et al. (1999) as an important inhibitory factor against BV and other pathogens. However, more recent studies show that H_2_O_2_ might not be effective for maintaining vaginal health (O'Hanlon et al., 2010, 2011; Gong et al., 2014). For example, O'Hanlon et al. (2010) did not observe any inhibitory activity of H_2_O_2_ against *G. vaginalis*, *P. bivia*, *Mycoplasma hominis, M. curtsii, Mobiluncus mulieris*, HSV-2, *N. gonorrhoeae* and *Hemophilus ducreyii*. The authors also observed that in order to have efficient H_2_O_2_ production *in vitro*, vigorous agitation to increase aeration is essential, which has been supported by other authors as well (Strus et al., 2006; Martin and Suarez, 2010). The low *in vivo* concentrations of H_2_O_2_ could therefore be explained by the relatively hypoxic environment of the vaginal lumen. Higher aeration and therefore production of H_2_O_2_ could possibly be achieved by sexual intercourse, although cervicovaginal fluid and semen have been shown to inactivate H_2_O_2_ (O'Hanlon et al., 2010; Gong et al., 2014). In a follow up study, the authors observed that under anaerobic growth conditions, physiological concentrations of lactic acid (55–111 mM) inactivated various BV-associated bacteria, while physiological concentrations of H_2_O_2_ (<100 μ M) showed no inactivation of the tested BV-associated bacteria, suggesting that lactic acid might be significantly more protective against infections in the vaginal environment in comparison to H_2_O_2_ (O'Hanlon et al., 2011). Moreover, at a concentration of 10 mM, H_2_O_2_ was more toxic to vaginal lactobacilli than to BV-associated bacteria (O'Hanlon et al., 2011). Of note, H_2_O_2_ has been investigated for clinical treatment of BV. For example, Cardone et al. (2003) showed that addition of 3% H_2_O_2_ for 1 week in the vaginal niche could eliminate the symptoms of BV and facilitate the restoration of a normal *Lactobacillus* dominated microbiota (Cardone et al., 2003). However, another study using single vaginal douching with 3% H_2_O_2_ failed to observe any positive results as compared to the metronidazole control group (Chaithongwongwatthana et al., 2003), indicating that H_2_O_2_ is not a key active metabolite of vaginal lactobacilli.

Therefore, the inhibition of *E. coli* and other BV-associated bacteria by *L. crispatus* is probably a result of lactic acid production or other not yet known factors. For example, Ravel et al. (2011) observed that *L*. *crispatus*-dominated communities have a lower vaginal pH as compared to communities dominated by other species, suggesting that *L*. *crispatus* is one of the highest producers of lactic acid, which is a key antimicrobial product of lactobacilli. Witkin et al. (2013) found that D-lactic acid, present in the highest amount in vaginal secretions of *L. crispatus*-dominated women, can inhibit extracellular matrix metalloproteinase inducer (EMMPRIN) production. EMMPRIN induces matrix metalloproteinase 8 (MMP-8), which is suggested to make the vaginal barrier more prone to upper genital infections linked to preterm birth. This might thus imply that D-lactic acid production is an important factor in the protection against preterm delivery. Moreover, *L. crispatus* can also have beneficial effects via immunomodulation. For example, *L*. *crispatus* ATCC 33820 was shown to inhibit *Candida albicans in vitro*, via modulation of Toll-like receptors (TLR) 2/4, interleukin 8 (IL-8) and human β-defensin 2 and 3 expression in epithelial cells (HeLa) (Rizzo et al., 2013). Taken together, these studies clearly suggest that the prevalence of *L*. *crispatus* in the VMB is an indicator of a healthy vaginal microbial ecosystem.

### *L. iners*—harmful or beneficial for vaginal health?

*L. iners* is one of the most commonly observed vaginal species, but was not commonly isolated since it is difficult to grow. It has been reported from both healthy and BV-diagnosed women, in contrast with *L. crispatus* which is mainly isolated from healthy women. The species was discovered by Falsen et al. (1999) and has escaped the attention of scientists for a long time, since it grows only on blood agar, but not on MRS or Rogosa (Falsen et al., 1999). *L. iners* has been detected in healthy Swedish women (determined by Nugent score), Canadian (with a normal Nugent score) (Burton and Reid, 2002), Nigerian (determined by Nugent score) (Anukam et al., 2006c), Brazilian (determined by Gram staining) (Martinez et al., 2008) and apparently healthy Chinese women (Shi et al., 2009). Ravel et al. (2011) showed that microbial communities belonging to group III (34.1%) of healthy white American and Asian women are dominated by *L. iners* as described above. Srinivasan and co-workers also reported that the VMB of 93% of American women without BV, as determined by Gram staining, are dominated by *L. iners* or *L. crispatus* (Srinivasan et al., 2012). However, the authors also observed that women with high levels of *L. iners* could be either BV positive or negative, demonstrating that the presence of *L. iners* as dominant *Lactobacillus* species might not be sufficient to protect against BV, in contrast to the conditions in which other *Lactobacillus* species dominate. Of note, other studies also detected *L. iners* during BV-associated conditions using different analysis methods (Burton and Reid, 2002; El Aila et al., 2009; Hummelen et al., 2010; Santiago et al., 2011). Interestingly, Witkin et al. (2013) showed that *L*. *iners*-dominated women have lower concentrations of D-lactic acid, suggesting that this could be one of the factors explaining the prevalence of BV in these women. This was also corroborated by their *in vitro* studies and genome analysis of *L*. *iners* which showed the incapability of the species to produce D-lactic acid (Witkin et al., 2013). Furthermore, *L. iners* has often been identified in the microbiota types intermediate between BV and normal microbiota. For example, *L. iners* was found to be dominant even after treatment of BV with metronidazole gel as studied by Ferris et al. (2007), and further supported by Jakobsson and Forsum (2007). The dominance of *L. iners* after treatment of BV (Srinivasan et al., 2012; Shipitsyna et al., 2013) led to the suggestion that *L. iners* might facilitate the transition between BV and non-BV states. It has been reported that a *L. iners* dominant state does not convert to a *L. crispatus*-dominated state even after BV treatment (Lambert et al., 2013). Additionally, *L. iners* was found to overgrow during menstruation, while the number of *L. crispatus* decreased (Srinivasan et al., 2010, 2012; Gajer et al., 2012; Santiago et al., 2012). Furthermore, Petricevic et al. (2014) observed that when *L*. *iners* was present alone without the presence of any other *Lactobacillus* species in pregnant women (*n* = 27), 40.7% of these women delivered preterm. Although the authors concluded on an association between solely *L. iners* presence in healthy pregnant women and preterm delivery, these results are not based on a well-controlled study population including 98 women who delivered at term and only 13 women who delivered preterm, requiring further studies.

Taking all data above together, the current data thus suggest that the presence of *L. iners* is not merely a biomarker reflecting a healthy human VMB. Of interest, the strain *L*. *iners* AB-1 has an unusually small genome of ~1.3 Mbp single chromosome, which seems to have undergone one or more rapid evolution events resulting in massive gene loss and acquisition of genes for optimal survival in the vaginal body site, such as iron-sulfur genes (Macklaim et al., 2011). The strain's genome encodes several genes that allow it to respond adequately to rapid changes in the environment, including CRISPR regions for phage resistance and genes for glycogen utilization, and maltose and mannose uptake. Interestingly, these genes appear to be over-expressed only during BV and not under normal healthy conditions (Macklaim et al., 2013). Additionally, the small genome size of *L. iners* may be indicative of a symbiotic or parasitic lifestyle in contrast to other lactobacilli that show niche flexibility and genome sizes of ca. 3 Mbp. Mendes-Soares et al. (2014) studied the genomes of several *L. iners* strains and found that all the strains lack several integral membrane proteins, protein families related to the acetyltransferase GNAT (Gcn5-related N-acetyltransferases) family and various transcriptional regulators present in other vaginal strains whereas they possess numerous ABC transporter permeases absent in other strains (Mendes-Soares et al., 2014). *L. iners* strains encode for inerolysin, a cholesterol-dependent pore-forming toxin, related to the vaginolysin virulence factor of *G. vaginalis* (Rampersaud et al., 2011). Therefore, further studies are needed to determine the exact role of this interesting species in vaginal health and disease and whether this strain is merely a biomarker of a vaginal microbiota in transition or could sometimes be a contributing factor to BV, as further discussed below.

### Non-*Lactobacillus* dominated healthy VMB

As mentioned above, recent studies also reported the presence of non-*Lactobacillus* dominated VMB in ca. 20–30% of healthy women (Zhou et al., 2010; Ravel et al., 2011; Srinivasan et al., 2012). These kind of vaginal communities are dominated by facultative or strict anaerobes, such as *Gardnerella*, *Corynebacterium*, *Atopobium, Anaerococcus, Prevotella*, *Peptoniphilus*, *Mobiluncus, Sneathia, Finegoldia*, and *Eggerthella*. Such diverse VMB appears to be typical for black and Hispanic women (Ravel et al., 2011). It is still debated whether this type of microbiota is thoroughly reflecting a healthy state or rather an asymptomatic state of BV. Previous studies suggested that even though this type of microbiota is non-lactobacilli dominated, the abundant species are able to maintain the crucial protective function of the vaginal niche, i.e. a low vaginal pH by production of lactic acid (Gajer et al., 2012). For example, members from *Atopobium*, *Streptococcus*, *Staphylococcus*, *Megasphaera*, and *Leptotrichia* are capable of homolactic or heterolactic acid fermentation (Zhou et al., 2004). Therefore, in the absence of symptoms, it appears that this type of diverse microbiota is normal and healthy. Nevertheless, a close and regular monitoring of women harboring a diverse type of VMB might be warranted to reduce the risk of BV, STIs or other problems in apparently healthy women, but who might have problems with fertility and preterm delivery.

### Temporal shifts in the composition of VMB

Most of the available studies on VMB are cross-sectional studies and thus only based on the collection of vaginal samples at a single time point. Nevertheless, the vaginal communities can change drastically over time based on changes in hormone levels, antibiotic treatments, sexual activities and/or hygiene practices. Therefore, a few recent prospective longitudinal studies examined the dynamics of the VMB, although mostly over a short period of time. For instance, the number of *L. iners* appears to remarkably increase during menses along with an increase of *G. vaginalis*, while they subsequently decrease after menses without intervention. Instead, the VMB dominated by *L. crispatus* appears to remain stable during menses (Srinivasan et al., 2010). The group of Vaneechoutte and co-workers reported that menses immensely disturbs the diversity of the VMB (Santiago et al., 2012). They observed a 100-fold decline in *L. crispatus* during menses, while the numbers of *L. iners, G. vaginalis, A. vaginae*, and *P. bivia* drastically increased in women with a normal microbiota (Santiago et al., 2012). They even reported that women with grade III VMB (diverse BV-associated state) had a more stable VMB than women with normal (grade I) microbiota. Gajer et al. (2012) also showed that the vaginal bacterial communities of some women markedly change over time, switching from one class to another, whereas others stay relatively stable. *L. crispatus*-dominated communities appear to often transform to a community state III dominated by *L. iners*, or to IV-A which is heterogeneous in composition and is characterized by a modest proportion of *L. crispatus*, *L. iners* or other *Lactobacillus* species as well as low numbers of strict anaerobic bacteria. *L. iners*-dominated communities shift more often to community type IV-B, which is dominated by a diverse number of bacteria belonging mainly to the genus *Atopobium*, *Prevotella*, *Parvimonas*, *Sneathia*, *Gardnerella*, or *Mobiluncus*, but in rare cases to IV-A. *L. gasseri*-dominated community groups rarely transit to other types and stay stable over time. The fluctuation of vaginal communities was affected by time in the menstruation cycle and to a certain extent by sexual activity (Gajer et al., 2012). Taken together, the studies of Srinivasan et al. (2010), Santiago et al. (2012) and Gajer et al. (2012) established (i) an important inter-individual variability in the VMB, (ii) a strong reduction of *L. crispatus* and its replacement by *L. iners* or by Gram positive cocci during menstruation, and (iii) long term stability of the VMB for only some women, which increases when *L. crispatus* is the dominant species. However, other studies show a different outcome. For instance, Chaban et al. (2014) did not observe any significant changes in the VMB during specific menstrual phases using *cpn60*-based analysis, although data of only 26 women were analyzed in this study. Nevertheless, all these studies on temporal shifts are in agreement with one of the major conclusions of the Human Microbiome Project that within-subject microbiota variation over time is lower than between-subject variation for all body sites, including the vagina (Human Microbiome Project Consortium, 2012).

## *Lactobacillus* species promote vaginal health by lowering the risk of BV

As yet introduced, one of the most common vaginal disorders is BV affecting fertile, premenopausal and pregnant women, resulting in millions of health care visits annually around the world. BV is a complex, polymicrobial disorder characterized by the disruption of the vaginal econiche, resulting in a reduction of lactobacilli and an overgrowth of strict or facultative anaerobic bacteria such as *Gardnerella* spp., *Atopobium* spp., *Prevotella* spp., *Mobiluncus* spp., as well as other taxa such as *Clostridium* spp., *Megasphaera* spp., *Leptotrichia* spp., and *Eggerthella*-like bacteria that were found even in pregnant women (Verstraelen et al., 2004; Fredricks et al., 2005; Tamrakar et al., 2007). Despite multiple attempts and focused research in the last decades to determine the exact cause of BV, the evidence is still poor. Multiple factors are reported to be involved in the development of BV, such as hormonal changes, the number of sexual partners, smoking, personal hygiene and antibiotic treatment (Hellberg et al., 2000; Verhelst et al., 2004; Brotman et al., 2008, 2014a). In recent years, culture-independent techniques based on the analysis of 16S rRNA gene sequences as described above have identified 3 phylogenetically-distinct DNA sequences representing potentially BV-associated bacteria (BVAB) BVAB1, BVAB2, and BVAB3, which are distantly related to the species of the phyla *Actinobacteria* and *Firmicutes*. BVABs, more specifically BVAB2, together with *Megasphaera*, *Leptotrichia*, and *Eggerthella*-like bacteria were found to be more representative species of BV than *Gardnerella* and *Atopobium* (Fredricks et al., 2005). Fluorescent *in-situ* hybridization (FISH) analysis by Swidsinski and coworkers revealed the presence of a dense polymicrobial biofilm on the vaginal epithelial surface in biopsies of women with BV. This biofilm is assumed to be initiated by *G*. *vaginalis* strains, which then becomes a scaffold for other species to adhere (Swidsinski et al., 2010; Verstraelen and Swidsinski, 2013). It is still uncertain whether these species are actually involved in the development of BV and therefore can be considered as pathogens, or if they are opportunistic organisms which are well adapted to the changes in the vaginal environment, as described for *L. iners* (see *L. iners*—harmful or beneficial for vaginal health?). When symptomatic, BV is characterized by vaginal discomfort and homogeneous malodorous vaginal discharge. Two methods are used for diagnosis of BV: the Amsel criteria and the Nugent scoring system. The Amsel criteria are often used in clinical practice (Amsel et al., 1983) and require the presence of at least three of the following criteria to diagnose BV: (i) thin, homogeneous vaginal discharge; (ii) vaginal pH higher than 4.5; (iii) “fishy” odor of vaginal fluid before or after addition of 10% potassium hydroxide (KOH) (whiff test); (iv) presence of clue cells on microscopic evaluation of saline wet preparations. However, the use of Amsel criteria is debatable, especially during pregnancy, when increased vaginal discharge is observed (Guise et al., 2001). There is not a major difference in the prevalence of vaginal discharge and odor among women with or without BV, which together with the fact that women often do not report any vaginal discharge, wetness, or odor makes the criteria subjective (Klebanoff et al., 2004). In addition, the pH value of the samples depends on how and where the samples are exactly taken. Secondly, the Nugent score is based on the Gram staining of vaginal smears and includes the microscopic quantitation of bacterial morphotypes yielding a score between 0 and 10 (Nugent et al., 1991). A score of 0–3 is normal, 4–6 is intermediate, and 7–10 is considered as BV. Important to mention is that around 50% of all women with BV as determined by Nugent score are asymptomatic. It is still unclear whether these women are asymptomatic or the symptoms are insignificantly pronounced and therefore poorly recognized and under-reported. These asymptomatic and most of the time non-inflammatory BV conditions can have important clinical consequences. For example, it has been reported that changes in the VMB are associated with various vaginal and urinary tract infections (Harmanli et al., 2000; Koumans et al., 2002). Furthermore, BV may result in increased rates of early pregnancy loss and preterm delivery (Eckert et al., 2003; Verstraelen et al., 2005). It has also been shown that BV facilitates the acquisition of sexually transmitted infections such as *Neisseria gonorrhoeae*, *Chlamydia trachomatis*, HIV and HSV-2 (Martin et al., 1999; Cherpes et al., 2003b; Wiesenfeld et al., 2003). Moreover, genital tract shedding of HIV, HSV-2 (Cherpes et al., 2005) and cytomegalovirus (Ross et al., 2005) is significantly higher in women with BV compared to BV-free women.

Several mechanisms have been proposed to explain how BV could increase the risk of STIs acquisition. First, the loss of protective *Lactobacillus* species and other changes in the vagina, such as elevated pH and decreased lactic acid concentrations, related to BV could facilitate the survival of vaginal pathogens. Secondly, BV-related microorganisms produce mucin-degrading enzymes in the vaginal fluid (e.g., sialidases), which degrade the mucus coating the vaginal and cervical epithelium, considered as one of the major components of the barrier against infection. Macklaim et al. (2013) also reported that the metabolome during BV conditions differs from the healthy stage. For example, the BV samples were enriched of enzymes, which belong mainly to *P. amnii* and some to *G. vaginalis*, used for the metabolism of glycans and more precisely of glycogen. The overexpression of those enzymes results in the production of succinate and short-chain fatty-acids, while healthy conditions are characterized by a high level of lactic acid. This mucus and glycogen degradation may cause micro-abrasions or epithelial cell alterations which could facilitate binding of pathogens to the underlying epithelial cell receptors. Finally, the immunological balance in the vaginal tract appears to be changed during BV with increased levels of pro-inflammatory cytokines which could render women more susceptible to the acquisition of STIs (Brotman, 2011; Gillet et al., 2011; Nardis et al., 2013).

Taken together, different studies on BV show different outcomes, which can be partially explained by the different diagnostic criteria used (e.g., Nugent score vs. Amsel criteria). There exists thus a clear need to complement these methods with culture-dependent and NGS methods to better diagnose BV and characterize the VMB.

## *Lactobacillus* species promote vaginal health by lowering the risk of STIs

STIs can be caused by over 30 bacterial, viral and parasitic pathogens known to be transmitted sexually, including vaginal, anal and oral sex. Important pathogens with a high incidence include bacterial pathogens such as *C. trachomatis*, *N. gonorrhoea* and viral pathogens such as HIV, HSV-2 and HPV. Since these infections have a considerable impact on reproductive and general health, STIs belong to the top five disease categories for which adults seek medical attention. Moreover, the majority of STIs remain asymptomatic which implies increased transmission risks of STIs. As described above, *Lactobacillus* species are thought to be a valuable biomarker for vaginal health supported by the fact that the presence of BV increases the risk of STIs acquisition. Therefore, a better understanding of the relationship between the vaginal microbiome and risk of STIs combined with refined biomarkers for a healthy VMB may lead to new strategies aiming at maintaining and/or restoring more protective vaginal bacterial communities.

### Bacterial STIs and the VMB

*C. trachomatis* infection is one of the most common bacterial STIs worldwide, with an estimated number of 105.7 million new infections annually in 2008. Untreated chlamydia may lead to pelvic inflammatory disease, tubal infertility and ectopic pregnancy. BV was found to be a strong predictor of *C. trachomatis* and *N. gonorrhoeae* infection among women with recent exposure to a male partner with chlamydial or gonococcal urethritis (Wiesenfeld et al., 2003). Another longitudinal study identified a significant association between intermediate/high Nugent score and increased risk of incident trichomonal, gonococcal and chlamydial infection (Brotman et al., 2010). Furthermore, longitudinal, randomized study observed that metronidazole treatment of BV-positive women for 1 year had significantly lower incidences of *Chlamydia*, however no appropriate placebo control was used (Schwebke and Desmond, 2007). Multiple studies thus suggest that BV increases the risk of incident *C. trachomatis* infection. Interestingly, combined 16S rRNA sequencing and metagenomics on vaginal samples of 101 *C. trachomatis*-infected women (92% African-American) treated at diagnosis revealed low proportions of *Lactobacillus* or specific *L. iners* genome types as a hallmark of the chlamydia infected state (Ma et al., 2013a).

*N. gonorrhoeae* is the second most common bacterial STIs affecting around 36.4 million adults worldwide in 2008. This Gram-negative bacterium causes infections of the female cervix, but also of the vagina, pharynx and rectum (Vielfort et al., 2008). As described above, multiple studies describe associations between BV and *N. gonorrhoea* infection. Given that the initial interaction between *N. gonorrhoeae* and the epithelial cells of the host is critical for successful colonization of the mucosa (Vielfort et al., 2008), several *in vitro* studies focused on the ability of vaginal lactobacilli to inhibit gonococcal adherence to epithelial cells. For example, it has been shown that *L. jensenii* ATCC 25258 could both reduce adhesion and invasion of *N. gonorrhoeae*, whereas *L. gasseri* ATCC 33323 could displace adherent *N. gonorrhoeae* (Spurbeck and Arvidson, 2008). A follow up study determined that released surface components (RSC) of *L. jensenii* ATCC 25258 are able to inhibit *N. gonorrhoeae* interaction with endometrial epithelial cells *in vitro* by occluding fibronectin binding sites. Future experiments are required to verify whether the surface-located enolase protein, identified as the main gonococcal adherence inhibiting factor of the RSC, is indeed the fibronectin-binding protein (Spurbeck and Arvidson, 2010). Nevertheless, future *in vivo* studies need to validate the importance of adherence competition in pathogen inhibition, in addition to more direct antimicrobial mechanisms of lactobacilli (see further).

### Viral STIs and the VMB

HIV infections remain one of the major global public health issues to date, accompanied by an estimated direct total lifetime medical cost of 12.6 billion dollars in the US in 2008 (Owusu-Edusei et al., 2013). By the end of 2013, approximately 35 million people were living with HIV and this number is increasing by around 2.1 million new infections each year. In order to increase the possibility for new therapies, a better understanding of the interplay between the VMB and HIV infections could be of crucial importance. A significant association was detected between women with BV, and thus lack of predominant *Lactobacillus* spp., and increased susceptibility to HIV acquisition (Atashili et al., 2008; Low et al., 2011). Furthermore, the prevalence and incidence of HSV-2 infections as well as the incidence of *N. gonorrhoeae* infections appear to be independent risk factors for HIV acquisition (van de Wijgert et al., 2009). Possible mechanisms for this relationship include (i) activation of immune response and inflammation during BV, (ii) disruption of the vaginal epithelium through which HIV could reach its target immune cells, and (iii) decreased *Lactobacillus* spp. causing elevated pH and lowered H_2_O_2_ concentrations (Petrova et al., 2013). Furthermore, BV is also often detected in HIV-seropositive women (Spear et al., 2011; Borgdorff et al., 2014). HIV-positive women with BV are characterized by a higher bacterial diversity compared to HIV-negative women with BV (Spear et al., 2008). Other studies also detected an association between BV and increased viral replication and HIV shedding suggesting that BV may have a role in enhancing HIV infectivity (Sha et al., 2005; Coleman et al., 2007; Tanton et al., 2011). Of note the presence of *G. vaginalis*, *M. hominis*, and *P. bivia* was also related to increased HIV expression whereas *L. acidophilus* was not (Hashemi et al., 2000). In addition, BV has been also associated with increased rates of transmission of HIV from infected women to their male partners, which suggests that BV could be responsible for new HIV-1 infections in Africa (Cohen et al., 2012). More detailed information on the antiviral mechanisms are discussed elsewhere (Petrova et al., 2013).

HSV-2, the main cause of genital herpes disease, is affecting around 20% of the female worldwide population in 2008 aged between 15 and 49 years (Looker et al., 2008). In order to better understand the relationship between the VMB and HSV-2 infections, multiple studies focused on the association between BV and HSV-2. Of note, the relationship between BV and HSV-2 is found to be potentially bidirectional. Several studies, some with longitudinal and some with cross-sectional study design, identified BV and the lack of *Lactobacillus*-predominant microbiota as an independent risk factor of HSV-2 acquisition (Cherpes et al., 2003a,b; Gottlieb et al., 2004; Kaul et al., 2007; Gallo et al., 2008). Moreover, a longitudinal study performed in the US described BV as a risk factor for HSV-2 replication and genital tract shedding, which can enhance the spread of HSV-2 (Cherpes et al., 2005). Evidence was also found that BV prevalence is higher after HSV-2 seroconversion in a female African population (Kaul et al., 2007; Nagot et al., 2007; Masese et al., 2014), for which Masese and colleagues described that incident HSV-2 was associated with a 30% increase in odds of episodes of BV. This association was also identified within female US populations (Allsworth et al., 2008; Cherpes et al., 2008). More specifically, positive HSV-2 serology is a risk factor for acquisition or subsequent episodes of BV (Cherpes et al., 2008; Masese et al., 2014). Prevalent HSV-2 infection was also associated with increased incidence of *Trichomonas vaginalis* and *N. gonorrhoeae* (Kaul et al., 2007). Of note, all studies mentioned above diagnosed BV by Nugent scoring and HSV-2 by PCR or serology testing. HSV-2 infection can also increase the rate of HIV-1 acquisition (Minton, 2013). To date, direct and indirect mechanisms could be responsible, with co-infection of cells by HIV and HSV-2 as a potential direct mechanism, which appeared unlikely based on infection results of an *ex vivo* skin explant model (Minton, 2013). Indirect mechanisms include disruption of epithelial barriers by HSV-2 and further inflammation, resulting in the recruitment of T lymphocytes and therefore increased episodes of HIV (Zhu et al., 2009). Furthermore, HSV-2 has also been shown to induce expression of an antimicrobial peptide LL-37 by keratinocytes, which is able to increase the susceptibility of Langerhans cells to HIV-1 due to increased expression levels of HIV coreceptors CD4 and CCR5 (Ogawa et al., 2013). Therefore, strategies to inhibit HSV-2 infection may also benefit indirect prevention of HIV-1 infections.

A third class of viral STIs which should not be underestimated consists of HPV infections. There are more than 100 types of HPV of which 13 are identified as high risk HPV types able to cause cervical cancer. Multiple studies have identified a positive association between BV and cervical HPV infection, supported by the overall estimated odds ratio of a meta-analysis including 12 eligible studies investigating the BV-HPV association (Gillet et al., 2011). BV was associated with the acquisition or reactivation of HPV infection, whereas no association was found with HPV persistence or the development of squamous intraepithelial lesions (SIL) (Watts et al., 2005). However, others observed a decreased or slower clearance of HPV lesions in women diagnosed with BV (Guo et al., 2012; Brotman et al., 2014b). More detailed molecular studies indicated that HPV infection seems to be associated with changes in the composition of the VMB, namely with a decrease in lactobacilli and increase in microbiota diversity (Clarke et al., 2012; Dols et al., 2012; Gao et al., 2013; Lee et al., 2013). These data are supported by the fact that a vaginal pH above 5 resulted in a 10–20% increased HPV risk and is associated with low-grade SIL diagnosis, as identified in premenopausal women from Costa Rica (Clarke et al., 2012). During HPV infection, *L. crispatus* was less frequently and *L. gasseri* and *G. vaginalis* were more frequently isolated from vaginal samples (Dols et al., 2012; Gao et al., 2013). A close study of a Korean twin cohort revealed an association between HPV and the abundance of Fusobacteria. In particular, *Sneathia* spp. were identified as potential microbiological markers of HPV infection, although the specificity for HPV should be further verified (Lee et al., 2013). Recently, a significant association between microbiota community types, as described by Gajer et al. (2012) using the same vaginal samples, and remission of HPV infection was observed. Apart from clusters III and IV having a greater proportion of HPV infections compared to other clusters, group IV-A increased the risk for transition to a HPV-positive state and group IV-B showed the slowest HPV clearance rate (Brotman et al., 2014b). In general, microbiota dominated by *L. iners* or lacking sufficient numbers of lactobacilli may represent microenvironments with increased risk of HPV acquisition/persistence (Brotman et al., 2014b). Taken together, reduced *Lactobacillus* counts are associated with HPV infection. Therefore, the rational selection of *Lactobacillus* probiotics with potential impact on HPV risk may represent a valuable strategy to lower HPV incidences/persistence. In a first longitudinal pilot study, 54 HPV-positive and low-grade SIL-positive women consumed a probiotic drink containing *L. casei* Shirota every day for 6 months. Probiotic users were twice as likely to have cervical lesion clearance, although no change in HPV detection was observed (Verhoeven et al., 2013). Further research is required to determine if probiotic interventions are able to reduce productive and/or recurrent HPV infections, as well as other viral STIs, since the current evidence is very limited.

Finally, we want to highlight the importance of well-designed clinical studies, which should include a statistically significant number of well-characterized women sampled longitudinally and which should control for potential confounding factors such as sociodemographic/economic, (sexual) behavioral and microbiological factors in order to define the exact role of the VMB in STIs, as well as to define the exact VMB communities. Primarily, attention should be given to the design of clinical studies examining associations between STIs and potential risk factors, which can be longitudinal or cross-sectional. Longitudinal studies, analyzing multiple samples per individual taken at follow up visits have the advantage to provide information on a causal relationship and possible fluctuations in STI episodes or VMB composition. Cross-sectional studies, analyzing only one sample per individual can only suggest association and should be taken within a representative time frame (e.g., not within menses, active/passive HSV-2 infection, etc.). Secondly, clinical data obtained should be adjusted for potential confounding factors of which important ones are age, race, sexual activity, condom use, number of sexual partners, time of menstruation, use of antibiotics, hormonal contraception, vaginal douching and BV/STI prevalence and episodes. For instance, it has been recently reported that use of condoms is associated with *L. crispatus* colonization of the vagina, which has also been shown to be protective against BV (as well as other STIs) (Ma et al., 2013b), indicating that it is an important confounding factor.

## How vaginal lactobacilli exert their health promoting effects

Since lactobacilli appear to be a hallmark of a healthy vaginal ecosystem, it is important to understand how they can exert important health-promoting effects, because knowledge on the exact molecular mediators can also promote the discovery and implementation of biomarkers. Postulated direct and indirect anti-pathogenic mechanisms of lactobacilli include (i) production of lactic acid and bacteriocins that directly kill or inhibit bacterial and viral pathogens, (ii) formation of microcolonies that adhere to the epithelial cell receptors and form a physical barrier against pathogen adhesion (Petrova et al., 2013), and (iii) stimulation of host defense mechanisms against pathogens (Lebeer et al., 2010). Yet again, although the health benefits of vaginal lactobacilli are widely recognized, they have been poorly substantiated by molecular studies.

### Production of lactic acid and bacteriocins

Directly linked to the presence of lactobacilli, the production of lactic acid is accepted as a hallmark beneficial activity of the VMB. Lactic acid has been linked to pathogen exclusion and their concentrations could also be seen as important biomarkers of vaginal health, although the current evidence is still mainly based on *in vitro* studies. For example, it has been shown that lactic acid is able to inactivate a wide range of reproductive tract pathogens, including the uropathogenic *E. coli* (Juarez Tomas et al., 2003), *N. gonorrhoeae* (Graver and Wade, 2011), and *C. trachomatis* (Gong et al., 2014). However, for the latter, similar inactivation was identified for formic acid and acetic acid, suggesting that sufficient hydrogen ions are involved in the mechanism to kill *Chlamydia* (Gong et al., 2014). Furthermore, a strong pH-independent inhibition of *C. trachomatis* by *L. brevis* CD2 (DSM 11988) could also be observed during early steps of infection. The same strain was also able to inhibit HSV-2-induced *Chlamydia* persistence (Mastromarino et al., 2014). Lactic acid has also been shown to play an important role in direct inactivation of HSV-2 (Conti et al., 2009) and HIV-1, due to effective capturing by acid cervicovaginal human mucus (Lai et al., 2009; Shukair et al., 2013). Given these data, lactic acid has been investigated for clinical treatment of BV. For example, re-establishment of the normal *Lactobacillus* dominated microbiota after the use of lactic acid gels has been reported for both pregnant and non-pregnant women after a few days of treatment (Andersch et al., 1986; Holst and Brandberg, 1990). However, another study failed to observe any positive results (Boeke et al., 1993), suggesting that well-designed clinical studies are required in order to investigate the possibility of using lactic acid as an alternative BV treatment.

Bacteriocins represent another main mechanism of direct inhibition of pathogens. Bacteriocins are known as ribosomally synthesized antimicrobial peptides and proteins with activity against closely related microorganisms (Cotter et al., 2013), although, as recently reported, several bacteriocins have a wide activity against unrelated microorganisms and viruses. For example, it has been shown that L23 bacteriocin (7 kDa) produced by *Lactobacillus fermentum* L23 displays a wide inhibitory activity against Gram-negative and Gram-positive pathogenic strains and *Candida* spp. (Pascual et al., 2008a). Fermenticin HV6b (class IIa antimicrobial peptide), produced by *L. fermentum* HV6b MTCC 10770, shows growth inhibition of *G. vaginalis*, *Mobiluncus*, staphylococci and streptococci (Kaur et al., 2013). In another study, Donia et al. (2014) were able to purify and reveal the structure of a thiopeptide antibiotic, named lactocillin, produced by reference strain *L. gasseri* JV-V03. Lactocillin has been shown to possess activity against *S. aureus*, *Enterococcus faecalis, G. vaginalis*, and *Corynebacterium aurimucosum* (Donia et al., 2014).

### Adhesion

Adherence of vaginal lactobacilli to host cells has been shown to prevent colonization by pathogenic microorganisms *in vitro* (Osset et al., 2001; Atassi et al., 2006) and *in vivo* (Anukam et al., 2006b). Hereby, the adherence pattern of lactobacilli appears to be more loose than that of (BV) pathogens that occur in thicker biofilms (Machado et al., 2013). Various cell surface components (proteins/glycoproteins, exopolysaccharides) of GI lactobacilli have been shown to play a role in bacterial adhesion to the host/pathogens (Lebeer et al., 2010). However, information on adhesins in vaginal *Lactobacillus* strains is lagging behind. Several vaginal *Lactobacillus* isolates have been shown to block the adhesion of vaginal pathogens to vaginal epithelial cells *in vitro*, such as by displacement and competition. For example, displacement of *G. vaginalis* by various lactobacilli has been reported (Mastromarino et al., 2002). Furthermore, exclusion and competition of *S. aureus*, Group B streptococci (Zarate and Nader-Macias, 2006), *Pseudomonas aeruginosa*, *Klebsiella pneumonia*, and *E. coli* (Osset et al., 2001), uropathogenic *E*. *coli* (UPEC), *G*. *vaginalis* and *P. bivia* (Atassi et al., 2006), and of *T. vaginalis* (Phukan et al., 2013) by *Lactobacillus* species have also been observed. An *in vitro* anti-HSV-2 activity of vaginal *L. brevis*, *L*. *plantarum*, and *L*. *salivarius* isolates was shown to be directly dependent on the adhesiveness of each bacterial strain to human epithelial cells (Mastromarino et al., 2002, 2011).

Interestingly, the genomes of the recently sequenced vaginal lactobacilli have been shown to encode various adhesins that could have a role in pathogen exclusion. For instance, the genome of *L*. *pentosus* KCA1 codes for large-sized mucus-binding and fibrinogen-binding proteins (FbpA) (Anukam et al., 2013). Recently, we were able to show that the genome of *L. plantarum* CMPG5300 contains many putative adhesion proteins, including predicted collagen-binding, mucus-binding, chitin-binding, fibrinogen-binding and mannose-binding proteins (Malik et al., 2014). The binding of *L*. *iners* AB-1 to fibrinogen has been suggested to be associated with the involvement of its genome-encoded fibrinogen-binding protein (McMillan et al., 2013), although this remains to be further validated, for example, by mutational analysis. A comparative genomic approach highlights that the strong adhesive capacity of *L. crispatus* and some unique adhesins play a key role in competitive exclusion of important BV pathogens such as *Gardnerella vaginalis* (Ojala et al., 2014). Although the exact role of specific adhesins in the adhesion of vaginal *Lactobacillus* species to host cells remains to be further explored, it becomes apparent that (loose) adherence of lactobacilli could be a biomarker of vaginal health. The rapid detection of specific *Lactobacillus* spp., such as *L. crispatus* detection in the human vagina by FISH approaches (Machado et al., 2013), could thus hold promise as novel molecular biomarkers of vaginal health.

### Stimulation of host defense mechanisms

Finally, lactobacilli probably also promote health in the vaginal ecosystem via various immunomodulation mechanisms, although this role is still poorly understood and mainly based on *in vitro* work. For example, Rose et al. (2012) reported that when the vaginal epithelial cells (VEC) were subjected to TLR agonists, *L. crispatus* ATCC 38820 and *L. jensenii* ATCC 25258 were able to temper the immune response. Furthermore, cytokine profiles of VEC were not affected after colonization with these commensal vaginal strains. However, the anti-inflammatory effect of the lactobacilli differed amongst agonists and strains, indicating that the immune-modulating effect of commensal strains depends on specific molecular interactions (Rose et al., 2012). This species-specific modulation of the host's immune response by the VMB was also inferred by Doerflinger et al. (2014), who discovered that *L. crispatus* ATCC 38820 does not significantly up-regulate pattern-recognition receptor (PRR) signaling pathways in human primary vaginal epithelial cells (V19), whereas *L. iners* ATCC 5195 does. Further, only the studied *L. iners* strain was shown to increase the expression of TNF at RNA level, although an increased secretion of TNF on protein level was not observed (Doerflinger et al., 2014). Whether *L. iners* is contributing to vaginal health is therefore still controversial. Wagner and Johnson (2012) investigated the influence of *L. rhamnosus* GR-1 and *L. reuteri* RC-14 on the inflammatory response of VK2/E6E7 to *C. albicans* and observed that the probiotic strains modulate the VK2/E6E7 inflammatory immune response to *C. albicans* by suppressing the expression of induced NF-κ B inhibitor kinase alpha (Iκ κ α), TLR2, TLR6, IL-8, and TNF, suggesting that they inhibit NF-κ B signaling (Wagner and Johnson, 2012). The effect of *L. rhamnosus* GR-1 and *L. reuteri* RC-14 on the host's immune system was also investigated *in vivo* (Bisanz et al., 2014). After treatment with these strains, significantly increased levels of IL-5 (as determined by cytokine ELISA assay) and IL-18 (as determined with microarray) could be detected in vaginal swabs, but not for IL-1β, TNF, IL-6, and GM-CSF. Furthermore, expression of complement receptors 1 and 3a and TLR2 was significantly increased, suggesting that the lactobacilli have instigated the innate immune system (Bisanz et al., 2014). Nevertheless, more research is needed to substantiate the expression of specific cytokines and immune factors as potential biomarkers of vaginal health.

## Probiotics—exogenously administered *Lactobacillus* strains to restore vaginal health

Since a healthy VMB is mainly dominated by *Lactobacillus* species, the perspective of using exogenously applied *Lactobacillus* probiotics to restore and/or maintain vaginal health becomes feasible. Probiotics are “live microorganisms that, when administered in adequate amounts, confer a health benefit on the host” (FAO/WHO, 2001). Various studies have yet been performed in order to investigate the role of single or a combination of probiotics for the treatment of BV as the most common vaginal disorder (Table 1) (the main focus of this review). Furthermore, several studies also showed the potential of exogenously administered probiotics for the treatment of another common vaginal disorder, namely candidiasis (Ehrstrom et al., 2010; Vicariotto et al., 2012; De et al., 2014).

**Table 1:** **Use of probiotic strains for the treatment of BV**.

**Type of intervention and probiotics used**	**BV cure rate**	**Type of study; Duration; Size**	**References**
**TREATMENT OF BV ONLY WITH PROBIOTICS**			
Vaginal capsules containing 10^8^ to 10^9^ CFU^*^ *L. acidophilus* or placebo for a period of 6 days	21% cure rate compared to 0% in the control group	R, DB, PC^**^; 20–40 days; 57 women	Hallen et al., 1992
Vaginal capsules introduced 1–2 times daily containing at least 10^7^ *L. acidophilus* and 0.03 mg estradiol for 6 days	88% cure rate compared to 22% in the control group	R, PC; 4 weeks; 32 women	Parent et al., 1996
Daily vaginal capsules containing 10^9^ CFU *L. rhamnosus* GR-1 and 10^9^ CFU *L. reuteri* RC-14 or twice a day 0.75% metronidazole gel for 5 days	65% cure rate compared to 33% in the metronidazole treated group	E, OB, AC; 30 days; 40 women	Anukam et al., 2006b
Vaginally introduced 1–2 capsules daily containing at least 10^9^ CFU of *L. brevis* CD2, *L. salivarius* FV2 and *L. plantarum* FV9 for a period of 7 days	50% cure rate compared to 6% in the control group	R, DB, PC; 3 weeks; 34 women	Mastromarino et al., 2009
**TREATMENT OF BV WITH PROBIOTICS IN COMBINATION WITH ANTIBIOTIC THERAPY**			
Vaginally introduced 100 mg clindamycin ovules for 3 days, subsequently tampons containing 10^8^ CFU of *L. gasseri*, *L. casei rhamnosus*, *L. fermentum* or placebo tampons during the next menstrual period	As defined by Amsel's criteria 56% cure rate in the probiotic group and 62%, in the control group; As defined by Nugent's score 55% cure rate in the probiotic group and 63%, cure in the control group	R, DB, PC; Two menstrual periods; 187 women	Eriksson et al., 2005
Oral intake of 500 mg metronidazole for 7 days and oral probiotic capsules containing *L. rhamnosus* GR-1 and *L. reuteri* RC-14 each 10^9^ CFU or placebo for 30 days starting on day 1 of the metronidazole treatment	88% cure rate compared to 40% in the control group	R, DB, PC; 30 days; 125 women	Anukam et al., 2006a
Oral intake of 300 mg clindamycin for 7 days, followed by vaginal capsules containing 10^9^ CFU *L. casei rhamnosus* for 7 days	83% cure rate compared to 35% in the control group	R, OB, PC; 4 weeks; 190 women	Petricevic and Witt, 2008
Vaginal cream containing 2% clindamycin followed by vaginal capsules containing 10^8^–10^9^ CFU *L. gasseri* Lba EB01-DSM 14869 and 10^8^–10^9^ *L. rhamnosus* Lbp PB01-DSM 14870. The probiotic treatment was performed and subsequently repeated for 10 days after each menstruation during 3 menstrual cycles	65% cure rate compared to 46% in the control group	R, DB, PC; 6 menstrual periods; 100 women	Larsson et al., 2008
Treatment with a single dose of tinidazole (2 g) plus either 2 oral capsules of *L. rhamnosus* GR-1 and *L. reuteri* RC-14 or placebo daily in the morning for 28 days, starting on the first day	The probiotic group had a significantly higher cure rate of BV (87.5%) than the placebo group (50.0%)	R, DB, PC; 28 days; 64 women	Martinez et al., 2009
Oral 500 mg of metronidazole followed by vaginal application once a week for 6 months of a capsule containing 40 mg of *L*. *rhamnosus* (N40000 CFU) beginning 8 days after the metronidazole therapy	During the first 6 months of follow-up, 96% of patients in the probiotic group had a balanced vaginal ecosystem. Follow-up over 12 months showed reduced recurrence of BV in the probiotic group	R, NB; 6 months; 46 women	Marcone et al., 2010
Oral intake of 400 mg metronidazole for period of 7 days followed by vaginal pessary containing *L. acidophilus* KS400 of 10^7^ CFU and 0.03 mg estriol for 12 days	72% cure rate compared to 73% in the control group	R, DB, PC; 6 months; 268 women	Bradshaw et al., 2012

* *CFU, colony forming units*.

** *R, randomized; DB, double blind; NB, not blind; PC, placebo controlled; OB, observer blind; AC, active controlled*.

### Treatment of BV using only probiotic therapy

One of the first studies to use probiotic strains for the treatment of BV was conducted in 1992 (Hallen et al., 1992). The authors used vaginal capsules containing 10^8^–10^9^ CFU of a H_2_O_2_ producing *L. acidophilus* (Vivag®, Pharma-Vinci A/S, Denmark) but did not observe any efficacy for the treatment of BV (as assessed by the Amsel criteria). Nevertheless, it is important to note that 50% of the patients in the probiotic group and 86% of the placebo group did not complete the trial, which makes it difficult to evaluate the efficacy of the study. In comparison, by using a H_2_O_2_ producing *L. acidophilus*, Parent et al. (1996) observed a high BV cure rate of 88% after 4 weeks of treatment. The authors conducted a placebo-controlled study that included both pregnant and non-pregnant women who were treated with a pharmaceutical product Gynoflor containing at least 10^7^ CFU *L. acidophilus* and 0.03 mg/ml estradiol (Parent et al., 1996). Both studies described above do not use well-characterized probiotic *Lactobacillus* strains. Even the species designation of these strains could be incorrect, since the group of *L. acidophilus* has been shown to be highly diverse (Falsen et al., 1999). The exact characterization of the probiotic strains used as well as their detailed molecular characterization is mandatory when conducting clinical trials and subsequently delivering a probiotic product to the market. The European Food Safety Authority (EFSA) has stated that an important step toward the approval of health claims for probiotic strains lies in better molecular research followed by multiple well-performed clinical trials. For example, two more recent clinical trials used well-characterized probiotic strains for the treatment of BV (Anukam et al., 2006b; Mastromarino et al., 2009) (Table 1). In the first study, *L. rhamnosus* GR-1 and *L. fermentum* RC-14 (now known as *L. reuteri* RC-14) were vaginally administrated (Anukam et al., 2006b). Both strains are well-known probiotic strains with well documented adhesion capacity to vaginal and uroepithelial cells, as well as capacity to inhibit adhesion and growth of uropathogens (Reid et al., 1987). In addition, *L. reuteri* RC-14 produces biosurfactant compounds (Velraeds et al., 1998) and significant amounts of H_2_O_2_ (Reid et al., 2001). The strains were also successfully recovered from the vaginal niche after oral administration and were shown to persist in the vaginal niche for 19 days post administration (Gardiner et al., 2002). Anukam et al. (2006b) compared the effect of intravaginally introduced capsules of *L. rhamnosus* GR-1 and *L. reuteri* RC-14 with metronidazole gel and reported a BV cure rate of 65% in the probiotic group in comparison to 33% in the metronidazole treated group (Anukam et al., 2006b). A few follow up studies with HIV patients showed that *L. rhamnosus* GR-1 and *L. reuteri* RC-14 were able to reduce episodes of diarrhea, nausea and flatulence (Anukam et al., 2008; Irvine et al., 2010) and to increase the CD4^+^ counts in several patients (Hummelen et al., 2011).

In addition to *L. rhamnosus* GR-1 and *L. reuteri* RC-14, others also used well-characterized and rationally selected vaginal *Lactobacillus* strains. For instance, an Italian group investigated capsules containing at least 10^9^ CFU of *L. brevis* CD2, *L. salivarius* subsp. *salicinius* FV2, and *L. plantarum* FV9 (Mastromarino et al., 2009) after intravaginal introduction using a commercially available product. As described above, the strains were reported to possess anti-pathogenic effect against *C. albicans*, *G. vaginalis*, HSV-2, and *C. trachomatis* (Mastromarino et al., 2002, 2011, 2014; Conti et al., 2009), and were able to colonize the human vagina (Massi et al., 2004). The authors observed that in the probiotic-treated group, the BV cure rate was 50% compared to only 6% in the placebo-treated group with the combined diagnostic methods (using Amsel criteria and Nugent scores), whereas a 67% vs. 12% cure rate was obtained when considering only the Amsel criteria (Mastromarino et al., 2009).

### Treatment of BV using probiotic therapy in combination with antibiotic therapy

Other studies investigate the role of probiotic treatment following standard antibiotic treatment (Table 1). The first study evaluates the BV cure rate after introducing freeze-dried strains of *L. gasseri*, *L. casei* subsp. *rhamnosus* and *L. fermentum* impregnated on tampons during menstruation (Eriksson et al., 2005). Each of the women received first clindamycin ovules vaginally once daily for 3 days, followed by probiotic treatment during the next menstruation period. However, the authors did not observe significant BV cure rates after the second menstruation in comparison to the control placebo group. The negative outcome might have multiple reasons, such as the number and the duration of the used tampons, the chosen strains, and the low concentration of *Lactobacillus* at the end of the study (only 10^6^ CFU/ml). Furthermore, as described above, the VMB drastically shifts during menstruation which can be the other possible explanation for the lack of effects observed by Eriksson et al. since the authors administrated the probiotics during menstruation. Anukam et al. (2006a) evaluated the augmentation of antimicrobial metronidazole therapy for BV by a 30-day oral probiotic treatment using *L. rhamnosus* GR-1 (10^9^ CFU/ml) and *L. reuteri* RC-14 (10^9^ CFU/ml). After 30-day treatment, 88% of the patients were cured in the antibiotic/probiotic group compared to 40% in the antibiotic/placebo group. The good record of *L. rhamnosus* GR-1 and *L. reuteri* RC-14 was also observed by Martinez et al. (2009) who used vaginal capsules containing the two strains in combination with a single dose of tinidazole (2 g). The authors observed that the probiotic group had a significantly higher cure rate of BV (87.5%) than the placebo group (50.0%). Additionally, according to the Nugent score, more women were assessed with normal VMB in the probiotic group (75.0 vs. 34.4% in the placebo group) (Martinez et al., 2009). Petricevic and Witt (2008) were also able to show restoration of the VMB after antibiotic treatment of BV and exogenously applied *L. casei* Lcr35 (10^9^ CFU/ml). Larsson et al. (2008) studied the effect of two probiotic strains introduced vaginally for 10 days during 3 menstrual cycles in a double blind, randomized, placebo-controlled trial. The lactobacilli used were commercially available as EcoVag® vaginal capsules (Bifodan A/S, Denmark) containing *L. gasseri* (Lba EB01-DSM 14869) with a concentration of 10^8−9^ CFU/capsule and *L. rhamnosus* (Lbp PB01-DSM 14870) with a minimal concentration of 10^8−9^ CFU/capsule. The study did not show a positive effect of the supplementary treatment with probiotic lactobacilli during the first month. However, a significantly reduced recurrence rate of BV at 6 months after the start of the treatment was observed (Larsson et al., 2008). Another more recent study also investigated the effect of probiotic treatment during a period of 6 months (Bradshaw et al., 2012). The authors used 10^7^ CFU/ml of live *L. acidophilus* KS400 introduced by a single vaginal pessary for 12 nights, but did not observe reduced BV recurrence. Finally, since *L. crispatus* species are often isolated from healthy vaginal niches, strains of this species hold great potential as probiotic, such as *L. crispatus* CTV-05 (LACIN-V), which appears safe in early clinical studies and is able to colonize the vaginal niche (Hemmerling et al., 2009, 2010).

It thus appears that only specific strains and treatment regimens show a beneficial effect of probiotics in BV treatment. Of note, the first conducted systematic review (only based on 4 studies) did not provide conclusive evidence that probiotics can enhance or are better than antibiotics in the treatment of BV (Senok et al., 2009). However, a more recent meta-analysis concluded that probiotics show a beneficial effect in patients who are suffering from BV, based on the included 12 clinical trials (summarized in Table 1) (Huang et al., 2014). One of the advantages of using probiotics unlike antibiotics is the fact that these beneficial bacteria can be used over a long period of time without interfering with the normal VMB. Therefore, probiotics cannot only be used for single treatment of BV but also for the prevention of BV recurrence in healthy women with a history of recurrent BV. For example, Ya et al. (2010) evaluated the effect of vaginal probiotic capsules (Probaclac Vaginal; Nicar Laboratories, Inc, Blainville, Quebec, Canada) on BV prophylaxis in healthy women with a history of recurrent BV in a randomized, placebo-controlled, double-blinded study. Between the 2- and 11-month follow up period, women who received probiotics reported lower recurrence rates of BV and *G*. *vaginalis* (Ya et al., 2010).

## Conclusions

Several culture-dependent and independent studies highlight the importance of vaginal lactobacilli for a healthy vaginal ecosystem. With the costs of molecular techniques steadily decreasing, it can be anticipated that molecular detection of the presence of lactobacilli at the species or even strain level could gain importance in clinical settings and might form important complementary approaches to the current Nugent and Amsel scoring methods, which could facilitate the use of lactobacilli as a vaginal health biomarker in the clinical setting. Over the last 10 years, NGS techniques have provided a more complete picture of the total bacterial diversity in the vaginal environment. Therefore, the definition of a healthy and normal vaginal microbiota has been re-considered. An emerging number of studies report the presence of non-*Lactobacillus* dominated VMBs, which potentially corresponds to so called asymptomatic BV, dominated by “BV-associated bacteria”. These asymptomatic cases of BV can explain why the treatment of recurrent BV is often unsuccessful. However, the same bacterial species have been also associated with the development of symptomatic BV, although the factors which trigger this condition are largely unknown. It is also questionable whether the “BV-associated bacteria” are really pathogenic and therefore possess pathogenic properties, or whether the host triggers environmental changes which activate different bacterial responses able to develop infection. The definition of a healthy and normal VMB is even more complicated when introducing STIs, which are clearly disease stages. Predisposition for many STIs has been directly linked to an unhealthy and abnormal microbiota, although STIs have also been associated with a healthy and normal VMB, suggesting that different types of microbiota might lead to different degrees of predisposition to STIs. Follow up studies are thus strongly required in order to understand the true role of the host and its microbiota in the process of STIs. Therefore, a healthy vaginal microbiota is not merely characterized by the presence of community types, but it is rather characterized by its beneficial function to the host and absence of symptoms, which can be provided by various vaginal types of microbiota, with or without lactobacilli.

Even if lactobacilli are not absolutely required for a healthy VMB, the fact that 70% of healthy women have a *Lactobacillus*-dominated microbiota, identified even using NGS techniques, highlights that the targeted and personalized application of *Lactobacillus*-based vaginal probiotics is still very valid. Successful administration of probiotics to the vaginal niche and treatment of BV will nevertheless depend on the exact *Lactobacillus* strain(s) used, applied dose, formulation, combination with standard antibiotic treatment, time of administration and duration of the treatment. To determine whether subjects are responders or non-responders to probiotic treatments, specific variables such as race, age of the subjects and dominant VMB might play key roles. Also, large-scale well-designed clinical trials with standardized protocols (consistency regarding species, dosage, route, timing and duration of administration) are of great importance to enable direct comparison between different probiotics.

### Conflict of interest statement

The authors declare that the research was conducted in the absence of any commercial or financial relationships that could be construed as a potential conflict of interest.

## References

[B1] Allsworth J. E. Lewis V. A. Peipert J. F. (2008). Viral sexually transmitted infections and bacterial vaginosis: 2001-2004 national health and nutrition examination survey data. Sex. Transm. Dis. 35, 791–796. 10.1097/OLQ.0b013e3181788301 18607314

[B1a] Amsel R. Totten P. A. Spiegel C. A. Chen K. C. Eschenbach D. Holmes K. K. (1983). Nonspecific vaginitis. Diagnostic criteria and microbial and epidemiologic associations. Am. J. Med. 74, 14–22. 6600371 10.1016/0002-9343(83)91112-9

[B2] Andersch B. Forssman L. Lincoln K. Torstensson P. (1986). Treatment of bacterial vaginosis with an acid cream: a comparison between the effect of lactate-gel and metronidazole. Gynecol. Obstet. Invest. 21, 19–25. 10.1159/000298923 3485071

[B2a] Antonio M. A. Hawes S. E. Hillier S. L. (1999). The identification of vaginal *Lactobacillus* species and the demographic and microbiologic characteristics of women colonized by these species. J. Infect. Dis. 180, 1950–1956. 10558952 10.1086/315109

[B3] Anukam K. C. Macklaim J. M. Gloor G. B. Reid G. Boekhorst J. Renckens B.. (2013). Genome sequence of *Lactobacillus pentosus* KCA1: vaginal isolate from a healthy premenopausal woman. PLoS ONE 8:e59239. 10.1371/journal.pone.0059239 23527145 PMC3602190

[B4] Anukam K. C. Osazuwa E. O. Ahonkhai I. Reid G. (2006c). *Lactobacillus* vaginal microbiota of women attending a reproductive health care service in Benin city, Nigeria. Sex Transm. Dis. 33, 59–62. 10.1097/01.olq.0000175367.15559.c4 16385223

[B5] Anukam K. C. Osazuwa E. O. Osadolor H. B. Bruce A. W. Reid G. (2008). Yogurt containing probiotic *Lactobacillus rhamnosus* GR-1 and *L. reuteri* RC-14 helps resolve moderate diarrhea and increases CD4 count in HIV/AIDS patients. J. Clin. Gastroenterol. 42, 239–243. 10.1097/MCG.0b013e31802c7465 18223503

[B6] Anukam K. C. Osazuwa E. Osemene G. I. Ehigiagbe F. Bruce A. W. Reid G. (2006b). Clinical study comparing probiotic *Lactobacillus* GR-1 and RC-14 with metronidazole vaginal gel to treat symptomatic bacterial vaginosis. Microbes. Infect. 8, 2772–2776. 10.1016/j.micinf.2006.08.008 17045832

[B7] Anukam K. Osazuwa E. Ahonkhai I. Ngwu M. Osemene G. Bruce A. W.. (2006a). Augmentation of antimicrobial metronidazole therapy of bacterial vaginosis with oral probiotic *Lactobacillus rhamnosus* GR-1 and *Lactobacillus reuteri* RC-14: randomized, double-blind, placebo controlled trial. Microbes. Infect. 8, 1450–1454. 10.1016/j.micinf.2006.01.003 16697231

[B8] Atashili J. Poole C. Ndumbe P. M. Adimora A. A. Smith J. S. (2008). Bacterial vaginosis and HIV acquisition: a meta-analysis of published studies. AIDS 22, 1493–1501. 10.1097/QAD.0b013e3283021a37 18614873 PMC2788489

[B9] Atassi F. Brassart D. Grob P. Graf F. Servin A. L. (2006). *Lactobacillus* strains isolated from the vaginal microbiota of healthy women inhibit *Prevotella bivia* and *Gardnerella vaginalis* in coculture and cell culture. FEMS Immunol. Med. Microbiol. 48, 424–432. 10.1111/j.1574-695X.2006.00162.x 17059467

[B10] Bisanz J. E. Seney S. McMillan A. Vongsa R. Koenig D. Wong L.. (2014). A systems biology approach investigating the effect of probiotics on the vaginal microbiome and host responses in a double blind, placebo-controlled clinical trial of post-menopausal women. PLoS ONE 9:e104511. 10.1371/journal.pone.0104511 25127240 PMC4134203

[B11] Boeke A. J. Dekker J. H. van Eijk J. T. Kostense P. J. Bezemer P. D. (1993). Effect of lactic acid suppositories compared with oral metronidazole and placebo in bacterial vaginosis: a randomised clinical trial. Genitourin. Med. 69, 388–392. 8244360 10.1136/sti.69.5.388 PMC1195125

[B12] Borgdorff H. Tsivtsivadze E. Verhelst R. Marzorati M. Jurriaans S. Ndayisaba G. F.. (2014). *Lactobacillus*-dominated cervicovaginal microbiota associated with reduced HIV/STI prevalence and genital HIV viral load in African women. ISME J. 8, 1781–1793. 10.1038/ismej.2014.26 24599071 PMC4139719

[B13] Bradshaw C. S. Pirotta M. De G. D. Hocking J. S. Morton A. N. Garland S. M.. (2012). Efficacy of oral metronidazole with vaginal clindamycin or vaginal probiotic for bacterial vaginosis: randomised placebo-controlled double-blind trial. PLoS ONE 7:e34540. 10.1371/journal.pone.0034540 22509319 PMC3317998

[B14] Brotman R. M. (2011). Vaginal microbiome and sexually transmitted infections: an epidemiologic perspective. J. Clin. Invest. 121, 4610–4617. 10.1172/JCI57172 22133886 PMC3225992

[B15] Brotman R. M. He X. Gajer P. Fadrosh D. Sharma E. Mongodin E. F.. (2014a). Association between cigarette smoking and the vaginal microbiota: a pilot study. BMC Infect. Dis. 14:471. 10.1186/1471-2334-14-471 25169082 PMC4161850

[B16] Brotman R. M. Klebanoff M. A. Nansel T. R. Andrews W. W. Schwebke J. R. Zhang J.. (2008). A longitudinal study of vaginal douching and bacterial vaginosis–a marginal structural modeling analysis. Am. J. Epidemiol. 168, 188–196. 10.1093/aje/kwn103 18503038 PMC2574994

[B17] Brotman R. M. Klebanoff M. A. Nansel T. R. Yu K. F. Andrews W. W. Zhang J. (2010). Bacterial vaginosis assessed by gram stain and diminished colonization resistance to incident gonococcal, chlamydial, and trichomonal genital infection. J. Infect. Dis. 202, 1907–1915 10.1086/657320 21067371 PMC3053135

[B18] Brotman R. M. Shardell M. D. Gajer P. Tracy J. K. Zenilman J. M. Ravel J.. (2014b). Interplay between the temporal dynamics of the vaginal microbiota and human papillomavirus detection. J. Infect. Dis. 210, 1723–1733. 10.1093/infdis/jiu330 24943724 PMC4296189

[B19] Burton J. P. Reid G. (2002). Evaluation of the bacterial vaginal flora of 20 postmenopausal women by direct (Nugent score) and molecular (polymerase chain reaction and denaturing gradient gel electrophoresis) techniques. J. Infect. Dis. 186, 1770–1780. 10.1086/345761 12447763

[B20] Cardone A. Zarcone R. Borrelli A. Di C. A. Russo A. Tartaglia E. (2003). Utilisation of hydrogen peroxide in the treatment of recurrent bacterial vaginosis. Minerva Ginecol. 55, 483–492. 14676737

[B21] Carey J. C. Klebanoff M. A. (2005). Is a change in the vaginal flora associated with an increased risk of preterm birth? Am. J. Obstet. Gynecol. 192, 1341–1346. 10.1016/j.ajog.2004.12.069 15846235

[B22] Chaban B. Links M. G. Jayaprakash T. P. Wagner E. C. Bourque D. K. Lohn Z.. (2014). Characterization of the vaginal microbiota of healthy Canadian women through the menstrual cycle. Microbiome 2:23. 10.1186/2049-2618-2-23 25053998 PMC4106219

[B23] Chaithongwongwatthana S. Limpongsanurak S. Sitthi-Amorn C. (2003). Single hydrogen peroxide vaginal douching versus single-dose oral metronidazole for the treatment of bacterial vaginosis: a randomized controlled trial. J. Med. Assoc. Thai. 86(Suppl. 2), S379–S384. 12930014

[B24] Cherpes T. L. Hillier S. L. Meyn L. A. Busch J. L. Krohn M. A. (2008). A delicate balance: risk factors for acquisition of bacterial vaginosis include sexual activity, absence of hydrogen peroxide-producing lactobacilli, black race, and positive herpes simplex virus type 2 serology. Sex Transm. Dis. 35, 78–83. 10.1097/OLQ.0b013e318156a5d0 17989585

[B25] Cherpes T. L. Melan M. A. Kant J. A. Cosentino L. A. Meyn L. A. Hillier S. L. (2005). Genital tract shedding of herpes simplex virus type 2 in women: effects of hormonal contraception, bacterial vaginosis, and vaginal group B *Streptococcus* colonization. Clin. Infect. Dis. 40, 1422–1428. 10.1086/429622 15844064

[B26] Cherpes T. L. Meyn L. A. Krohn M. A. Hillier S. L. (2003a). Risk factors for infection with herpes simplex virus type 2: role of smoking, douching, uncircumcised males, and vaginal flora. Sex Transm. Dis. 30, 405–410. 10.1097/00007435-200305000-00006 12916131

[B27] Cherpes T. L. Meyn L. A. Krohn M. A. Lurie J. G. Hillier S. L. (2003b). Association between acquisition of herpes simplex virus type 2 in women and bacterial vaginosis. Clin. Infect. Dis. 37, 319–325. 10.1086/375819 12884154

[B28] Clarke M. A. Rodriguez A. C. Gage J. C. Herrero R. Hildesheim A. Wacholder S.. (2012). A large, population-based study of age-related associations between vaginal pH and human papillomavirus infection. BMC Infect. Dis. 12:33. 10.1186/1471-2334-12-33 22316377 PMC3292496

[B29] Cohen C. R. Lingappa J. R. Baeten J. M. Ngayo M. O. Spiegel C. A. Hong T.. (2012). Bacterial vaginosis associated with increased risk of female-to-male HIV-1 transmission: a prospective cohort analysis among African couples. PLoS Med. 9:e1001251. 10.1371/journal.pmed.1001251 22745608 PMC3383741

[B30] Coleman J. S. Hitti J. Bukusi E. A. Mwachari C. Muliro A. Nguti R.. (2007). Infectious correlates of HIV-1 shedding in the female upper and lower genital tracts. AIDS 21, 755–759. 10.1097/QAD.0b013e328012b838 17413697

[B31] Conti C. Malacrino C. Mastromarino P. (2009). Inhibition of herpes simplex virus type 2 by vaginal lactobacilli. J. Physiol. Pharmacol. 60(Suppl. 6), 19–26. 20224147

[B32] Cotter P. D. Ross R. P. Hill C. (2013). Bacteriocins - a viable alternative to antibiotics? Nat. Rev. Microbiol. 11, 95–105. 10.1038/nrmicro2937 23268227

[B33] Damelin L. H. Paximadis M. Mavri-Damelin D. Birkhead M. Lewis D. A. Tiemessen C. T. (2011). Identification of predominant culturable vaginal *Lactobacillus* species and associated bacteriophages from women with and without vaginal discharge syndrome in South Africa. J. Med. Microbiol. 60, 180–183. 10.1099/jmm.0.024463-0 21030503

[B34] Datcu R. Gesink D. Mulvad G. Montgomery-Andersen R. Rink E. Koch A.. (2013). Vaginal microbiome in women from Greenland assessed by microscopy and quantitative PCR. BMC Infect. Dis. 13:480. 10.1186/1471-2334-13-480 24131550 PMC3853076

[B35] De S. F. Parazzini F. De L. R. Banco R. Maso G. P. De S. D.. (2014). Lactobacillus plantarum P17630 for preventing Candida vaginitis recurrence: a retrospective comparative study. Eur. J. Obstet. Gynecol. Reprod. Biol. 182, 136–139. 10.1016/j.ejogrb.2014.09.018 25305660

[B36] Doerflinger S. Y. Throop A. L. Herbst-Kralovetz M. M. (2014). Bacteria in the vaginal microbiome alter the innate immune response and barrier properties of the human vaginal epithelia in a species-specific manner. J. Infect. Dis. 209, 1989–1999. 10.1093/infdis/jiu004 24403560

[B37] Dols J. A. Reid G. Kort R. Schuren F. H. Tempelman H. Bontekoe T. R.. (2012). PCR-based identification of eight *Lactobacillus* species and 18 hr-HPV genotypes in fixed cervical samples of South African women at risk of HIV and BV. Diagn. Cytopathol. 40, 472–477. 10.1002/dc.21786 22021225

[B38] Dominguez-Bello M. G. Costello E. K. Contreras M. Magris M. Hidalgo G. Fierer N.. (2010). Delivery mode shapes the acquisition and structure of the initial microbiota across multiple body habitats in newborns. Proc. Natl. Acad. Sci. U.S.A. 107, 11971–11975. 10.1073/pnas.1002601107 20566857 PMC2900693

[B39] Donders G. G. Van C. K. Bellen G. Reybrouck R. Van den B. T. Riphagen I.. (2009). Predictive value for preterm birth of abnormal vaginal flora, bacterial vaginosis and aerobic vaginitis during the first trimester of pregnancy. BJOG 116, 1315–1324. 10.1111/j.1471-0528.2009.02237.x 19538417

[B40] Donia M. S. Cimermancic P. Schulze C. J. Wieland Brown L. C. Martin J. Mitreva M.. (2014). A systematic analysis of biosynthetic gene clusters in the human microbiome reveals a common family of antibiotics. Cell 158, 1402–1414. 10.1016/j.cell.2014.08.032 25215495 PMC4164201

[B41] Drell T. Lillsaar T. Tummeleht L. Simm J. Aaspollu A. Vain E.. (2013). Characterization of the vaginal micro- and mycobiome in asymptomatic reproductive-age Estonian women. PLoS ONE 8:e54379. 10.1371/journal.pone.0054379 23372716 PMC3553157

[B41a] Eckert L. O. Moore D. E. Patton D. L. Agnew K. J. Eschenbach D. A. (2003). Relationship of vaginal bacteria and inflammation with conception and early pregnancy loss following *in-vitro* fertilization. Infect. Dis Obstet. Gynecol. 11, 11–17. 10.1155/S1064744903000024 12839628 PMC1852261

[B42] Ehrstrom S. Daroczy K. Rylander E. Samuelsson C. Johannesson U. Anzen B.. (2010). Lactic acid bacteria colonization and clinical outcome after probiotic supplementation in conventionally treated bacterial vaginosis and vulvovaginal candidiasis. Microbes. Infect. 12, 691–699. 10.1016/j.micinf.2010.04.010 20472091

[B43] El Aila N. A. Tency I. Claeys G. Verstraelen H. Saerens B. Santiago G. L.. (2009). Identification and genotyping of bacteria from paired vaginal and rectal samples from pregnant women indicates similarity between vaginal and rectal microflora. BMC Infect. Dis. 9:167. 10.1186/1471-2334-9-167 19828036 PMC2770471

[B44] Eriksson K. Carlsson B. Forsum U. Larsson P. G. (2005). A double-blind treatment study of bacterial vaginosis with normal vaginal lactobacilli after an open treatment with vaginal clindamycin ovules. Acta. Derm. Venereol. 85, 42–46. 10.1080/00015550410022249 15848990

[B45] Falsen E. Pascual C. Sjoden B. Ohlen M. Collins M. D. (1999). Phenotypic and phylogenetic characterization of a novel *Lactobacillus* species from human sources: description of *Lactobacillus iners* sp. nov. Int. J. Syst. Bacteriol. 49(Pt 1), 217–221. 10.1099/00207713-49-1-217 10028266

[B45a] Ferris M. J. Norori J. Zozaya-Hinchliffe M. Martin D. H. (2007). Cultivation-independent analysis of changes in bacterial vaginosis flora following metronidazole treatment. J. Clin. Microbiol. 45, 1016–1018. 10.1128/JCM.02085-06 17202272 PMC1829144

[B46] Forney L. J. Gajer P. Williams C. J. Schneider G. M. Koenig S. S. McCulle S. L.. (2010). Comparison of self-collected and physician-collected vaginal swabs for microbiome analysis. J. Clin. Microbiol. 48, 1741–1748. 10.1128/JCM.01710-09 20200290 PMC2863907

[B47] Fredricks D. N. Fiedler T. L. Marrazzo J. M. (2005). Molecular identification of bacteria associated with bacterial vaginosis. N. Engl. J. Med. 353, 1899–1911. 10.1056/NEJMoa043802 16267321

[B48] Gajer P. Brotman R. M. Bai G. Sakamoto J. Schutte U. M. Zhong X.. (2012). Temporal dynamics of the human vaginal microbiota. Sci. Transl. Med. 4, 132ra52. 10.1126/scitranslmed.3003605 22553250 PMC3722878

[B49] Gallo M. F. Warner L. Macaluso M. Stone K. M. Brill I. Fleenor M. E.. (2008). Risk factors for incident herpes simplex type 2 virus infection among women attending a sexually transmitted disease clinic. Sex Transm. Dis. 35, 679–685. 10.1097/OLQ.0b013e31816fcaf8 18461012

[B50] Gao W. Weng J. Gao Y. Chen X. (2013). Comparison of the vaginal microbiota diversity of women with and without human papillomavirus infection: a cross-sectional study. BMC Infect. Dis. 13:271. 10.1186/1471-2334-13-271 23758857 PMC3684509

[B51] Gardiner G. E. Heinemann C. Bruce A. W. Beuerman D. Reid G. (2002). Persistence of *Lactobacillus fermentum* RC-14 and *Lactobacillus rhamnosus* GR-1 but not *L. rhamnosus* GG in the human vagina as demonstrated by randomly amplified polymorphic DNA. Clin. Diagn. Lab. Immunol. 9, 92–96 10.1128/CDLI.9.1.92-96.2002 11777835 PMC119863

[B52] Ghartey J. P. Smith B. C. Chen Z. Buckley N. Lo Y. Ratner A. J.. (2014). Lactobacillus crispatus dominant vaginal microbiome is associated with inhibitory activity of female genital tract secretions against *Escherichia coli*. PLoS ONE 9:e96659. 10.1371/journal.pone.0096659 24805362 PMC4013016

[B53] Gillet E. Meys J. F. Verstraelen H. Bosire C. De S. P. Temmerman M.. (2011). Bacterial vaginosis is associated with uterine cervical human papillomavirus infection: a meta-analysis. BMC Infect. Dis. 11:10. 10.1186/1471-2334-11-10 21223574 PMC3023697

[B54] Gong Z. Luna Y. Yu P. Fan H. (2014). Lactobacilli inactivate *Chlamydia trachomatis* through lactic acid but not H_2_O_2_. PLoS ONE 9:e107758. 10.1371/journal.pone.0107758 25215504 PMC4162611

[B55] Gottlieb S. L. Douglas J. M. Jr. Foster M. Schmid D. S. Newman D. R. Baron A. E.. (2004). Incidence of herpes simplex virus type 2 infection in 5 sexually transmitted disease (STD) clinics and the effect of HIV/STD risk-reduction counseling. J. Infect. Dis. 190, 1059–1067. 10.1086/423323 15319854

[B56] Graver M. A. Wade J. J. (2011). The role of acidification in the inhibition of *Neisseria gonorrhoeae* by vaginal lactobacilli during anaerobic growth. Ann. Clin. Microbiol. Antimicrob. 10:8. 10.1186/1476-0711-10-8 21329492 PMC3045876

[B56a] Guise J. M. Mahon S. Aickin M. Helfand M. Peipert J. Westhoff C. (2001). Screening for bacterial vaginosis in pregnancy. Am. J. Prev. Med. 20, 62–67. 11306234 10.1016/s0749-3797(01)00256-2

[B57] Guo Y. L. You K. Qiao J. Zhao Y. M. Geng L. (2012). Bacterial vaginosis is conducive to the persistence of HPV infection. Int. J. STD AIDS 23, 581–584. 10.1258/ijsa.2012.011342 22930296

[B58] Gustafsson R. J. Ahrne S. Jeppsson B. Benoni C. Olsson C. Stjernquist M.. (2011). The *Lactobacillus* flora in vagina and rectum of fertile and postmenopausal healthy Swedish women. BMC Womens Health 11, 17–11. 10.1186/1472-6874-11-17 21609500 PMC3118184

[B59] Hallen A. Jarstrand C. Pahlson C. (1992). Treatment of bacterial vaginosis with lactobacilli. Sex Transm. Dis. 19, 146–148. 10.1097/00007435-199205000-00007 1523530

[B59a] Harmanli O. H. Cheng G. Y. Nyirjesy P. Chatwani A. Gaughan J. P. (2000). Urinary tract infections in women with bacterial vaginosis. Obstet. Gynecol. 95, 710–712. 10.1016/S0029-7844(99)00632-8 10775734

[B60] Hashemi F. B. Ghassemi M. Faro S. Aroutcheva A. Spear G. T. (2000). Induction of human immunodeficiency virus type 1 expression by anaerobes associated with bacterial vaginosis. J. Infect. Dis. 181, 1574–1580. 10.1086/315455 10823756

[B61] Hellberg D. Nilsson S. Mardh P. A. (2000). Bacterial vaginosis and smoking. Int. J. STD AIDS 11, 603–606. 10.1258/0956462001916461 10997505

[B62] Hemmerling A. Harrison W. Schroeder A. Park J. Korn A. Shiboski S.. (2009). Phase 1 dose-ranging safety trial of *Lactobacillus crispatus* CTV-05 for the prevention of bacterial vaginosis. Sex Transm. Dis. 36, 564–569. 10.1097/OLQ.0b013e3181a74924 19543144 PMC2758081

[B63] Hemmerling A. Harrison W. Schroeder A. Park J. Korn A. Shiboski S.. (2010). Phase 2a study assessing colonization efficiency, safety, and acceptability of *Lactobacillus crispatus* CTV-05 in women with bacterial vaginosis. Sex Transm. Dis. 37, 745–750. 10.1097/OLQ.0b013e3181e50026 20644497

[B64] Holst E. Brandberg A. (1990). Treatment of bacterial vaginosis in pregnancy with a lactate gel. Scand. J. Infect. Dis. 22, 625–626. 10.3109/00365549009027109 2259873

[B65] Huang H. Song L. Zhao W. (2014). Effects of probiotics for the treatment of bacterial vaginosis in adult women: a meta-analysis of randomized clinical trials. Arch. Gynecol. Obstet. 289, 1225–1234. 10.1007/s00404-013-3117-0 24318276

[B66] Human Microbiome Project Consortium (2012). Structure, function and diversity of the healthy human microbiome. Nature 486, 207–214. 10.1038/nature11234 22699609 PMC3564958

[B67] Hummelen R. Changalucha J. Butamanya N. L. Koyama T. E. Cook A. Habbema J. D.. (2011). Effect of 25 weeks probiotic supplementation on immune function of HIV patients. Gut. Microbes. 2, 80–85. 10.4161/gmic.2.2.15787 21637031

[B68] Hummelen R. Fernandes A. D. Macklaim J. M. Dickson R. J. Changalucha J. Gloor G. B.. (2010). Deep sequencing of the vaginal microbiota of women with HIV. PLoS ONE 5:e12078. 10.1371/journal.pone.0012078 20711427 PMC2920804

[B69] Irvine S. L. Hummelen R. Hekmat S. Looman C. W. Habbema J. D. Reid G. (2010). Probiotic yogurt consumption is associated with an increase of CD4 count among people living with HIV/AIDS. J. Clin. Gastroenterol. 44, e201–e205. 10.1097/MCG.0b013e3181d8fba8 20463586

[B69a] Jakobsson T. Forsum U. (2007). Lactobacillus iners: a marker of changes in the vaginal flora? J. Clin. Microbiol. 45, 3145. 17652481 10.1128/JCM.00558-07 PMC2045263

[B70] Jespers V. Menten J. Smet H. Poradosu S. Abdellati S. Verhelst R.. (2012). Quantification of bacterial species of the vaginal microbiome in different groups of women, using nucleic acid amplification tests. BMC Microbiol. 12:83. 10.1186/1471-2180-12-83 22647069 PMC3418157

[B71] Juarez Tomas M. S. Ocana V. S. Wiese B. Nader-Macias M. E. (2003). Growth and lactic acid production by vaginal *Lactobacillus acidophilus* CRL 1259, and inhibition of uropathogenic *Escherichia coli*. J. Med. Microbiol. 52, 1117–1124. 10.1099/jmm.0.05155-0 14614071

[B72] Kaul R. Nagelkerke N. J. Kimani J. Ngugi E. Bwayo J. J. Macdonald K. S.. (2007). Prevalent herpes simplex virus type 2 infection is associated with altered vaginal flora and an increased susceptibility to multiple sexually transmitted infections. J. Infect. Dis. 196, 1692–1697. 10.1086/522006 18008255

[B73] Kaur B. Balgir P. P. Mittu B. Kumar B. Garg N. (2013). Biomedical applications of fermenticin HV6b isolated from *Lactobacillus fermentum* HV6b MTCC10770. Biomed. Res. Int. 168438. 10.1155/2013/168438 23984320 PMC3745898

[B74] Kilic A. O. Pavlova S. I. Alpay S. Kilic S. S. Tao L. (2001). Comparative study of vaginal *Lactobacillus* phages isolated from women in the United States and Turkey: prevalence, morphology, host range, and DNA homology. Clin. Diagn. Lab. Immunol. 8, 31–39. 10.1128/CDLI.8.1.31-39.2001 11139192 PMC96007

[B75] Kiss H. Kogler B. Petricevic L. Sauerzapf I. Klayraung S. Domig K.. (2007). Vaginal *Lactobacillus* microbiota of healthy women in the late first trimester of pregnancy. BJOG 114, 1402–1407. 10.1111/j.1471-0528.2007.01412.x 17877778

[B76] Klebanoff M. A. Schwebke J. R. Zhang J. Nansel T. R. Yu K. F. Andrews W. W. (2004). Vulvovaginal symptoms in women with bacterial vaginosis. Obstet. Gynecol. 104, 267–272. 10.1097/01.AOG.0000134783.98382.b0 15291998

[B76a] Koumans E. H. Markowitz L. E. Hogan V. (2002). Indications for therapy and treatment recommendations for bacterial vaginosis in nonpregnant and pregnant women: a synthesis of data. Clin. Infect. Dis. 35(Suppl. 2), S152–S172. 10.1086/342103 12353202

[B77] Lai S. K. Hida K. Shukair S. Wang Y. Y. Figueiredo A. Cone R.. (2009). Human immunodeficiency virus type 1 is trapped by acidic but not by neutralized human cervicovaginal mucus. J. Virol. 83, 11196–11200. 10.1128/JVI.01899-08 19692470 PMC2772788

[B78] Lambert J. A. John S. Sobel J. D. Akins R. A. (2013). Longitudinal analysis of vaginal microbiome dynamics in women with recurrent bacterial vaginosis: recognition of the conversion process. PLoS ONE 8:e82599. 10.1371/journal.pone.0082599 24376552 PMC3869700

[B79] Larsson P. G. Stray-Pedersen B. Ryttig K. R. Larsen S. (2008). Human lactobacilli as supplementation of clindamycin to patients with bacterial vaginosis reduce the recurrence rate; a 6-month, double-blind, randomized, placebo-controlled study. BMC Womens Health 8:3. 10.1186/1472-6874-8-3 18197974 PMC3225869

[B80] Lebeer S. Vanderleyden J. De Keersmaecker S. C. (2010). Host interactions of probiotic bacterial surface molecules: comparison with commensals and pathogens. Nat. Rev. Microbiol. 8, 171–184. 10.1038/nrmicro2297 20157338

[B81] Lee J. E. Lee S. Lee H. Song Y. M. Lee K. Han M. J.. (2013). Association of the vaginal microbiota with human papillomavirus infection in a Korean twin cohort. PLoS ONE 8:e63514. 10.1371/journal.pone.0063514 23717441 PMC3661536

[B82] Looker K. J. Garnett G. P. Schmid G. P. (2008). An estimate of the global prevalence and incidence of herpes simplex virus type 2 infection. Bull. World Health Organ. 86, 805–812. 10.2471/BLT.07.046128 18949218 PMC2649511

[B83] Low N. Chersich M. F. Schmidlin K. Egger M. Francis S. C. van de Wijgert J. H.. (2011). Intravaginal practices, bacterial vaginosis, and HIV infection in women: individual participant data meta-analysis. PLoS Med. 8:e1000416. 10.1371/journal.pmed.1000416 21358808 PMC3039685

[B84] Ma B. Brotman R. M. Gajer P. Fadrosh D. Mahurkar A. White O.. (2013a). Association between chlamydia trachomatis genital infection and the vaginal microbiome. Sex Transm. Infect. 89:A35. 10.1136/sextrans-2013-051184.011025668647

[B85] Ma L. Lv Z. Su J. Wang J. Yan D. Wei J.. (2013b). Consistent condom use increases the colonization of Lactobacillus crispatus in the vagina. PLoS ONE 8:e70716. 10.1371/journal.pone.0070716 23894682 PMC3720897

[B86] Machado A. Jefferson K. K. Cerca N. (2013). Interactions between Lactobacillus crispatus and Bacterial Vaginosis (BV)-associated bacterial species in initial attachment and biofilm formation. Int. J. Mol. Sci. 14, 12004–12012. 10.3390/ijms140612004 23739678 PMC3709769

[B87] Macklaim J. M. Fernandes A. D. Di Bella J. M. Hammond J. A. Reid G. Gloor G. B. (2013). Comparative meta-RNA-seq of the vaginal microbiota and differential expression by *Lactobacillus iners* in health and dysbiosis. Microbiome 1:12. 10.1186/2049-2618-1-12 24450540 PMC3971606

[B88] Macklaim J. M. Gloor G. B. Anukam K. C. Cribby S. Reid G. (2011). At the crossroads of vaginal health and disease, the genome sequence of *Lactobacillus iners* AB-1. Proc. Natl. Acad. Sci. U.S.A. 108, 4688–4695. 10.1073/pnas.1000086107 21059957 PMC3063587

[B89] Malik S. Siezen R. J. Renckens B. Vaneechoutte M. Vanderleyden J. Lebeer S. (2014). Draft genome sequence of Lactobacillus plantarum CMPG5300, a human vaginal isolate. Genome Announc. 2, e01149–e01114. 10.1128/genomeA.01149-14 25395634 PMC4241660

[B90] Marcone V. Rocca G. Lichtner M. Calzolari E. (2010). Long-term vaginal administration of *Lactobacillus rhamnosus* as a complementary approach to management of bacterial vaginosis. Int. J. Gynaecol. Obstet. 110, 223–226. 10.1016/j.ijgo.2010.04.025 20573348

[B92a] Martin H. L. Richardson B. A. Nyange P. M. Lavreys L. Hillier S. L. Chohan B. (1999). Vaginal lactobacilli, microbial flora, and risk of human immunodeficiency virus type 1 and sexually transmitted disease acquisition. J. Infect. Dis. 180, 1863–1868. 10558942 10.1086/315127

[B92] Martin D. H. Zozaya M. Lillis R. Miller J. Ferris M. J. (2012). The microbiota of the human genitourinary tract: trying to see the forest through the trees. Trans. Am. Clin. Climatol. Assoc. 123, 242–256. 23303991 PMC3540603

[B93] Martin R. Soberon N. Vaneechoutte M. Florez A. B. Vazquez F. Suarez J. E. (2008). Characterization of indigenous vaginal lactobacilli from healthy women as probiotic candidates. Int. Microbiol. 11, 261–266. 10.2436/20.1501.01.70 19204898

[B94] Martin R. Suarez J. E. (2010). Biosynthesis and degradation of H2O2 by vaginal lactobacilli. Appl. Environ. Microbiol. 76, 400–405. 10.1128/AEM.01631-09 19948869 PMC2805228

[B95] Martinez R. C. Franceschini S. A. Patta M. C. Quintana S. M. Gomes B. C. De Martinis E. C.. (2009). Improved cure of bacterial vaginosis with single dose of tinidazole (2 g), Lactobacillus rhamnosus GR-1, and Lactobacillus reuteri RC-14: a randomized, double-blind, placebo-controlled trial. Can. J. Microbiol. 55, 133–138. 10.1139/W08-102 19295645

[B96] Martinez R. C. Franceschini S. A. Patta M. C. Quintana S. M. Nunes A. C. Moreira J. L.. (2008). Analysis of vaginal lactobacilli from healthy and infected Brazilian women. Appl. Environ. Microbiol. 74, 4539–4542. 10.1128/AEM.00284-08 18502927 PMC2493183

[B97] Martinez-Pena M. D. Castro-Escarpulli G. guilera-Arreola M. G. (2013). Lactobacillus species isolated from vaginal secretions of healthy and bacterial vaginosis-intermediate Mexican women: a prospective study. BMC Infect. Dis. 13:189. 10.1186/1471-2334-13-189 23617246 PMC3655868

[B98] Masese L. Baeten J. M. Richardson B. A. Bukusi E. John-Stewart G. Jaoko W.. (2014). Incident herpes simplex virus type 2 infection increases the risk of subsequent episodes of bacterial vaginosis. J. Infect. Dis. 209, 1023–1027. 10.1093/infdis/jit634 24273042 PMC3952675

[B99] Massi M. Vitali B. Federici F. Matteuzzi D. Brigidi P. (2004). Identification method based on PCR combined with automated ribotyping for tracking probiotic Lactobacillus strains colonizing the human gut and vagina. J. Appl. Microbiol. 96, 777–786. 10.1111/j.1365-2672.2004.02228.x 15012816

[B100] Mastromarino P. Brigidi P. Macchia S. Maggi L. Pirovano F. Trinchieri V.. (2002). Characterization and selection of vaginal Lactobacillus strains for the preparation of vaginal tablets. J. Appl. Microbiol. 93, 884–893. 10.1046/j.1365-2672.2002.01759.x 12392537

[B101] Mastromarino P. Cacciotti F. Masci A. Mosca L. (2011). Antiviral activity of Lactobacillus brevis towards herpes simplex virus type 2: role of cell wall associated components. Anaerobe 17, 334–336. 10.1016/j.anaerobe.2011.04.022 21621625

[B102] Mastromarino P. Di P. M. Schiavoni G. Nardis C. Gentile M. Sessa R. (2014). Effects of vaginal lactobacilli in Chlamydia trachomatis infection. Int. J. Med. Microbiol. 304, 654–661. 10.1016/j.ijmm.2014.04.006 24875405

[B103] Mastromarino P. Macchia S. Meggiorini L. Trinchieri V. Mosca L. Perluigi M.. (2009). Effectiveness of Lactobacillus-containing vaginal tablets in the treatment of symptomatic bacterial vaginosis. Clin. Microbiol Infect. 15, 67–74. 10.1111/j.1469-0691.2008.02112.x 19046169

[B104] McMillan A. Macklaim J. M. Burton J. P. Reid G. (2013). Adhesion of Lactobacillus iners AB-1 to Human fibronectin: a key mediator for persistence in the vagina? Reprod. Sci. 20, 791–796. 10.1177/1933719112466306 23202727

[B105] Mendes-Soares H. Suzuki H. Hickey R. J. Forney L. J. (2014). Comparative functional genomics of Lactobacillus spp. Reveals possible mechanisms for specialization of vaginal lactobacilli to their environment. J. Bacteriol. 196, 1458–1470. 10.1128/JB.01439-13 24488312 PMC3993339

[B106] Minton K. (2013). HIV: from one STI to another… with love. Nat. Rev. Immunol. 13, 154–155. 10.1038/nri3402 23391993

[B107] Mitchell C. Balkus J. E. Fredricks D. Liu C. Kernan-Mullin J. Frenkel L. M.. (2013). Interaction between lactobacilli, bacterial vaginosis-associated bacteria, and HIV Type 1 RNA and DNA genital shedding in U.S. and Kenyan women. AIDS Res. Hum. Retroviruses 29, 13–19. 10.1089/AID.2012.0187 23020644 PMC3537306

[B108] Nagot N. Ouedraogo A. Defer M. C. Vallo R. Mayaud P. Van de P. P. (2007). Association between bacterial vaginosis and Herpes simplex virus type-2 infection: implications for HIV acquisition studies. Sex Transm. Infect. 83, 365–368. 10.1136/sti.2007.024794 17493979 PMC2659027

[B109] Nardis C. Mosca L. Mastromarino P. (2013). Vaginal microbiota and viral sexually transmitted diseases. Ann. Ig. 25, 443–456. 10.7416/ai.2013 24048183

[B109a] Nugent R. P. Krohn M. A. Hillier S. L. (1991). Reliability of diagnosing bacterial vaginosis is improved by a standardized method of gram stain interpretation. J. Clin. Microbiol. 29, 297–301. 1706728 10.1128/jcm.29.2.297-301.1991 PMC269757

[B110] Ogawa Y. Kawamura T. Matsuzawa T. Aoki R. Gee P. Yamashita A.. (2013). Antimicrobial peptide LL-37 produced by HSV-2-infected keratinocytes enhances HIV infection of Langerhans cells. Cell Host. Microbe. 13, 77–86. 10.1016/j.chom.2012.12.002 23332157

[B111] O'Hanlon D. E. Lanier B. R. Moench T. R. Cone R. A. (2010). Cervicovaginal fluid and semen block the microbicidal activity of hydrogen peroxide produced by vaginal lactobacilli. BMC Infect. Dis. 10:120. 10.1186/1471-2334-10-120 20482854 PMC2887447

[B112] O'Hanlon D. E. Moench T. R. Cone R. A. (2011). In vaginal fluid, bacteria associated with bacterial vaginosis can be suppressed with lactic acid but not hydrogen peroxide. BMC Infect. Dis. 11:200. 10.1186/1471-2334-11-200 21771337 PMC3161885

[B113] Ojala T. Kankainen M. Castro J. Cerca N. Edelman S. Westerlund-Wikstrom B.. (2014). Comparative genomics of Lactobacillus crispatus suggests novel mechanisms for the competitive exclusion of Gardnerella vaginalis. BMC Genomics 15:1070. 10.1186/1471-2164-15-1070 25480015 PMC4300991

[B114] Osset J. Bartolome R. M. Garcia E. Andreu A. (2001). Assessment of the capacity of Lactobacillus to inhibit the growth of uropathogens and block their adhesion to vaginal epithelial cells. J. Infect. Dis. 183, 485–491. 10.1086/318070 11133381

[B115] Owusu-Edusei K. Jr. Chesson H. W. Gift T. L. Tao G. Mahajan R. Ocfemia M. C.. (2013). The estimated direct medical cost of selected sexually transmitted infections in the United States, 2008. Sex Transm. Dis. 40, 197–201. 10.1097/OLQ.0b013e318285c6d2 23403600

[B116] Parent D. Bossens M. Bayot D. Kirkpatrick C. Graf F. Wilkinson F. E.. (1996). Therapy of bacterial vaginosis using exogenously-applied Lactobacilli acidophili and a low dose of estriol: a placebo-controlled multicentric clinical trial. Arzneimittelforschung 46, 68–73. 8821521

[B117] Pascual L. M. Daniele M. B. Giordano W. Pajaro M. C. Barberis I. L. (2008a). Purification and partial characterization of novel bacteriocin L23 produced by *Lactobacillus fermentum* L23. Curr. Microbiol. 56, 397–402. 10.1007/s00284-007-9094-4 18172715

[B118] Pascual L. M. Daniele M. B. Ruiz F. Giordano W. Pajaro C. Barberis L. (2008b). *Lactobacillus rhamnosus* L60, a potential probiotic isolated from the human vagina. J. Gen. Appl. Microbiol. 54, 141–148. 10.2323/jgam.54.141 18654035

[B119] Petricevic L. Domig K. J. Nierscher F. J. Sandhofer M. J. Fidesser M. Krondorfer I.. (2014). Characterisation of the vaginal *Lactobacillus* microbiota associated with preterm delivery. Sci. Rep. 4:5136. 10.1038/srep05136 24875844 PMC4038809

[B120] Petricevic L. Witt A. (2008). The role of *Lactobacillus casei rhamnosus* Lcr35 in restoring the normal vaginal flora after antibiotic treatment of bacterial vaginosis. BJOG 115, 1369–1374. 10.1111/j.1471-0528.2008.01882.x 18823487

[B121] Petrova M. I. van den B. M. Balzarini J. Vanderleyden J. Lebeer S. (2013). Vaginal microbiota and its role in HIV transmission and infection. FEMS Microbiol Rev. 37, 762–792. 10.1111/1574-6976.12029 23789590

[B122] Phukan N. Parsamand T. Brooks A. E. Nguyen T. N. Simoes-Barbosa A. (2013). The adherence of *Trichomonas vaginalis* to host ectocervical cells is influenced by lactobacilli. Sex Transm. Infect. 89, 455–459. 10.1136/sextrans-2013-051039 23720602

[B123] Rampersaud R. Planet P. J. Randis T. M. Kulkarni R. Aguilar J. L. Lehrer R. I.. (2011). Inerolysin, a cholesterol-dependent cytolysin produced by Lactobacillus iners. J. Bacteriol. 193, 1034–1041. 10.1128/JB.00694-10 21169489 PMC3067590

[B124] Ravel J. Gajer P. Abdo Z. Schneider G. M. Koenig S. S. McCulle S. L.. (2011). Vaginal microbiome of reproductive-age women. Proc. Natl. Acad. Sci. U.S.A. 1, 4680–4687. 10.1073/pnas.1002611107 20534435 PMC3063603

[B125a] Reid G. Bruce A. W. Fraser N. Heinemann C. Owen J. Henning B. (2001). Oral probiotics can resolve urogenital infections. FEMS Immunol. Med. Microbiol. 30, 49–52. 10.1111/j.1574-695X.2001.tb01549.x 11172991

[B125b] Reid G. Cook R. L. Bruce A. W. (1987). Examination of strains of lactobacilli for properties that may influence bacterial interference in the urinary tract. J. Urol. 138, 330–335. 3599250 10.1016/s0022-5347(17)43137-5

[B125] Rizzo A. Losacco A. Carratelli C. R. (2013). *Lactobacillus crispatus* modulates epithelial cell defense against *Candida albicans* through Toll-like receptors 2 and 4, interleukin 8 and human beta-defensins 2 and 3. Immunol. Lett. 156, 102–109. 10.1016/j.imlet.2013.08.013 24120511

[B126a] Ross S. A. Novak Z. Ashrith G. Rivera L. B. Britt W. J. Hedges S.. (2005). Association between genital tract cytomegalovirus infection and bacterial vaginosis. J. Infect. Dis. 192, 1727–1730. 10.1086/497150 16235170

[B126] Rose W. A. McGowin C. L. Spagnuolo R. A. Eaves-Pyles T. D. Popov V. L. Pyles R. B. (2012). Commensal bacteria modulate innate immune responses of vaginal epithelial cell multilayer cultures. PLoS ONE 7:e32728. 10.1371/journal.pone.0032728 22412914 PMC3296736

[B127] Santiago G. L. Cools P. Verstraelen H. Trog M. Missine G. El A. N.. (2011). Longitudinal study of the dynamics of vaginal microflora during two consecutive menstrual cycles. PLoS ONE 6:e28180. 10.1371/journal.pone.0028180 22140538 PMC3227645

[B128] Santiago G. L. Tency I. Verstraelen H. Verhelst R. Trog M. Temmerman M.. (2012). Longitudinal qPCR study of the dynamics of *L. crispatus, L. iners, A. vaginae*, (sialidase positive) *G. vaginalis, and P. bivia in the vagina*. PLoS ONE 7:e45281. 10.1371/journal.pone.0045281 23028904 PMC3448655

[B129] Schwebke J. R. Desmond R. (2007). A randomized trial of metronidazole in asymptomatic bacterial vaginosis to prevent the acquisition of sexually transmitted diseases. Am. J. Obstet. Gynecol. 196, 517–516. 10.1016/j.ajog.2007.02.048 17547876 PMC1993882

[B130] SenokA. C.VerstraelenH.TemmermanM.BottaG. A. (2009). Probiotics for the treatment of bacterial vaginosis. Cochrane. Database. Syst. Rev. 4:CD006289. 10.1002/14651858.CD006289.pub219821358

[B131] Sha B. E. Zariffard M. R. Wang Q. J. Chen H. Y. Bremer J. Cohen M. H.. (2005). Female genital-tract HIV load correlates inversely with *Lactobacillus* species but positively with bacterial vaginosis and *Mycoplasma hominis*. J. Infect. Dis. 191, 25–32. 10.1086/426394 15592999

[B132] Shi Y. Chen L. Tong J. Xu C. (2009). Preliminary characterization of vaginal microbiota in healthy Chinese women using cultivation-independent methods. J. Obstet. Gynaecol. Res. 35, 525–532. 10.1111/j.1447-0756.2008.00971.x 19527394

[B133] Shipitsyna E. Roos A. Datcu R. Hallen A. Fredlund H. Jensen J. S.. (2013). Composition of the vaginal microbiota in women of reproductive age–sensitive and specific molecular diagnosis of bacterial vaginosis is possible? PLoS ONE 8:e60670. 10.1371/journal.pone.0060670 23585843 PMC3621988

[B134] Shukair S. A. Allen S. A. Cianci G. C. Stieh D. J. Anderson M. R. Baig S. M.. (2013). Human cervicovaginal mucus contains an activity that hinders HIV-1 movement. Mucosal. Immunol. 6, 427–434. 10.1038/mi.2012.87 22990624 PMC3732193

[B135] Siezen R. J. Kleerebezem M. (2011). The human gut microbiome: are we our enterotypes? Microb. Biotechnol. 4, 550–553. 10.1111/j.1751-7915.2011.00290.x 21848611 PMC3819005

[B136] Smith B. C. McAndrew T. Chen Z. Harari A. Barris D. M. Viswanathan S.. (2012). The cervical microbiome over 7 years and a comparison of methodologies for its characterization. PLoS ONE 7:e40425. 10.1371/journal.pone.0040425 22792313 PMC3392218

[B137] Spear G. T. Gilbert D. Landay A. L. Zariffard R. French A. L. Patel P.. (2011). Pyrosequencing of the genital microbiotas of HIV-seropositive and -seronegative women reveals *Lactobacillus iners* as the predominant *Lactobacillus* Species. Appl. Environ. Microbiol. 77, 378–381. 10.1128/AEM.00973-10 21075899 PMC3019699

[B138] Spear G. T. Sikaroodi M. Zariffard M. R. Landay A. L. French A. L. Gillevet P. M. (2008). Comparison of the diversity of the vaginal microbiota in HIV-infected and HIV-uninfected women with or without bacterial vaginosis. J. Infect. Dis. 198, 1131–1140. 10.1086/591942 18717638 PMC2800037

[B139] Spurbeck R. R. Arvidson C. G. (2008). Inhibition of *Neisseria gonorrhoeae* epithelial cell interactions by vaginal *Lactobacillus* species. Infect. Immun. 76, 3124–3130. 10.1128/IAI.00101-08 18411284 PMC2446695

[B140] Spurbeck R. R. Arvidson C. G. (2010). *Lactobacillus jensenii* surface-associated proteins inhibit *Neisseria gonorrhoeae* adherence to epithelial cells. Infect. Immun. 78, 3103–3111. 10.1128/IAI.01200-09 20385752 PMC2897381

[B141] Srinivasan S. Hoffman N. G. Morgan M. T. Matsen F. A. Fiedler T. L. Hall R. W.. (2012). Bacterial communities in women with bacterial vaginosis: high resolution phylogenetic analyses reveal relationships of microbiota to clinical criteria. PLoS ONE 7:e37818. 10.1371/journal.pone.0037818 22719852 PMC3377712

[B142] Srinivasan S. Liu C. Mitchell C. M. Fiedler T. L. Thomas K. K. Agnew K. J.. (2010). Temporal variability of human vaginal bacteria and relationship with bacterial vaginosis. PLoS ONE 5:e10197. 10.1371/journal.pone.0010197 20419168 PMC2855365

[B143] Strus M. Brzychczy-Wloch M. Gosiewski T. Kochan P. Heczko P. B. (2006). The *in vitro* effect of hydrogen peroxide on vaginal microbial communities. FEMS Immunol. Med. Microbiol. 48, 56–63. 10.1111/j.1574-695X.2006.00120.x 16965352

[B144] Swidsinski A. Doerffel Y. Loening-Baucke V. Swidsinski S. Verstraelen H. Vaneechoutte M.. (2010). *Gardnerella* biofilm involves females and males and is transmitted sexually. Gynecol. Obstet. Invest. 70, 256–263. 10.1159/000314015 21051845

[B145] Tamrakar R. Yamada T. Furuta I. Cho K. Morikawa M. Yamada H.. (2007). Association between *Lactobacillus* species and bacterial vaginosis-related bacteria, and bacterial vaginosis scores in pregnant Japanese women. BMC Infect. Dis. 7:128. 10.1186/1471-2334-7-128 17986357 PMC2212641

[B146] Tanton C. Weiss H. A. Le G. J. Changalucha J. Rusizoka M. Baisley K.. (2011). Correlates of HIV-1 genital shedding in Tanzanian women. PLoS ONE 6:e17480. 10.1371/journal.pone.0017480 21390251 PMC3046975

[B147a] van de Wijgert J. H. Borgdorff H. Verhelst R. Crucitti T. Francis S. Verstraelen H. (2014). The vaginal microbiota: what have we learned after a decade of molecular characterization? PLoS ONE 9:e105998. 25148517 10.1371/journal.pone.0105998 PMC4141851

[B147] van de Wijgert J. H. Morrison C. S. Brown J. Kwok C. Van der P. B. Chipato T.. (2009). Disentangling contributions of reproductive tract infections to HIV acquisition in African Women. Sex Transm. Dis. 36, 357–364. 10.1097/OLQ.0b013e3181a4f695 19434010

[B148] Vasquez A. Jakobsson T. Ahrne S. Forsum U. Molin G. (2002). Vaginal lactobacillus flora of healthy Swedish women. J. Clin. Microbiol. 40, 2746–2749. 10.1128/JCM.40.8.2746-2749.2002 12149323 PMC120688

[B149a] Velraeds M. M. van de Belt-Gritter B. van der Mei H. C. Reid G. Busscher H. J. (1998). Interference in initial adhesion of uropathogenic bacteria and yeasts to silicone rubber by a *Lactobacillus* acidophilus biosurfactant. J. Med. Microbiol. 47, 1081–1085. 9856644 10.1099/00222615-47-12-1081

[B149] Verhelst R. Verstraelen H. Claeys G. Verschraegen G. Delanghe J. Van S. L.. (2004). Cloning of 16S rRNA genes amplified from normal and disturbed vaginal microflora suggests a strong association between *Atopobium vaginae, Gardnerella vaginalis* and bacterial vaginosis. BMC Microbiol. 4:16. 10.1186/1471-2180-4-16 15102329 PMC419343

[B150] Verhelst R. Verstraelen H. Claeys G. Verschraegen G. Van S. L. De G. C.. (2005). Comparison between Gram stain and culture for the characterization of vaginal microflora: definition of a distinct grade that resembles grade I microflora and revised categorization of grade I microflora. BMC Microbiol. 5:61. 10.1186/1471-2180-5-61 16225680 PMC1266370

[B151] Verhoeven V. Renard N. Makar A. Van R. P. Bogers J. P. Lardon F.. (2013). Probiotics enhance the clearance of human papillomavirus-related cervical lesions: a prospective controlled pilot study. Eur. J. Cancer Prev. 22, 46–51. 10.1097/CEJ.0b013e328355ed23 22706167

[B152a] Verstraelen H. Delanghe J. Roelens K. Blot S. Claeys G. Temmerman M. (2005). Subclinical iron deficiency is a strong predictor of bacterial vaginosis in early pregnancy. BMC Infect. Dis. 5:55. 10.1186/1471-2334-5-55 16000177 PMC1199597

[B152] Verstraelen H. Swidsinski A. (2013). The biofilm in bacterial vaginosis: implications for epidemiology, diagnosis and treatment. Curr. Opin. Infect. Dis. 26, 86–89. 10.1097/QCO.0b013e32835c20cd 23221767

[B153] Verstraelen H. Verhelst R. Claeys G. Temmerman M. Vaneechoutte M. (2004). Culture-independent analysis of vaginal microflora: the unrecognized association of *Atopobium vaginae* with bacterial vaginosis. Am. J. Obstet. Gynecol. 191, 1130–1132. 10.1016/j.ajog.2004.04.013 15507931

[B154] Vicariotto F. Del P. M. Mogna L. Mogna G. (2012). Effectiveness of the association of 2 probiotic strains formulated in a slow release vaginal product, in women affected by vulvovaginal candidiasis: a pilot study. J. Clin. Gastroenterol. 10.1097/MCG.0b013e3182684d71 22955364

[B155] Vielfort K. Sjolinder H. Roos S. Jonsson H. Aro H. (2008). Adherence of clinically isolated lactobacilli to human cervical cells in competition with Neisseria gonorrhoeae. Microbes. Infect. 10, 1325–1334. 10.1016/j.micinf.2008.07.032 18761100

[B156] Wagner R. D. Johnson S. J. (2012). Probiotic lactobacillus and estrogen effects on vaginal epithelial gene expression responses to *Candida albicans*. J. Biomed. Sci. 19:58. 10.1186/1423-0127-19-58 22715972 PMC3404894

[B157] Watts D. H. Fazzari M. Minkoff H. Hillier S. L. Sha B. Glesby M.. (2005). Effects of bacterial vaginosis and other genital infections on the natural history of human papillomavirus infection in HIV-1-infected and high-risk HIV-1-uninfected women. J. Infect. Dis. 191, 1129–1139. 10.1086/427777 15747249

[B158] Wiesenfeld H. C. Hillier S. L. Krohn M. A. Landers D. V. Sweet R. L. (2003). Bacterial vaginosis is a strong predictor of Neisseria gonorrhoeae and Chlamydia trachomatis infection. Clin. Infect. Dis. 36, 663–668. 10.1086/367658 12594649

[B159] Witkin S. S. Mendes-Soares H. Linhares I. M. Jayaram A. Ledger W. J. Forney L. J. (2013). Influence of vaginal bacteria and D- and L-lactic acid isomers on vaginal extracellular matrix metalloproteinase inducer: implications for protection against upper genital tract infections. MBio 4, e00460–e00413. 10.1128/mBio.00460-13 23919998 PMC3735189

[B160] Ya W. Reifer C. Miller L. E. (2010). Efficacy of vaginal probiotic capsules for recurrent bacterial vaginosis: a double-blind, randomized, placebo-controlled study. Am. J. Obstet. Gynecol. 203, 120–126. 10.1016/j.ajog.2010.05.023 20659602

[B161] Zarate G. Nader-Macias M. E. (2006). Influence of probiotic vaginal lactobacilli on *in vitro* adhesion of urogenital pathogens to vaginal epithelial cells. Lett. Appl. Microbiol. 43, 174–180. 10.1111/j.1472-765X.2006.01934.x 16869901

[B162] Zhou X. Bent S. J. Schneider M. G. Davis C. C. Islam M. R. Forney L. J. (2004). Characterization of vaginal microbial communities in adult healthy women using cultivation-independent methods. Microbiology 150, 2565–2573. 10.1099/mic.0.26905-0 15289553

[B163] Zhou X. Hansmann M. A. Davis C. C. Suzuki H. Brown C. J. Schutte U. (2010). The vaginal bacterial communities of Japanese women resemble those of women in other racial groups. FEMS Immunol. Med. Microbiol. 58, 169–181 10.1111/j.1574-695X.2009.00618.x 19912342 PMC2868947

[B164] Zhu J. Hladik F. Woodward A. Klock A. Peng T. Johnston C.. (2009). Persistence of HIV-1 receptor-positive cells after HSV-2 reactivation is a potential mechanism for increased HIV-1 acquisition. Nat. Med. 15, 886–892. 10.1038/nm.2006 19648930 PMC2723183

